# Contignasterines, Anti-Inflammatory 2‑Aminoimidazole Steroids from the Sponge *Neopetrosia* cf. *rava* Collected in the Bismarck Sea

**DOI:** 10.1021/acs.jnatprod.5c00118

**Published:** 2025-03-07

**Authors:** Juan Ortega-Vidal, Maggie M. Reddy, Nadia Pérez-Fuentes, Rebeca Alvariño, Svenja Burth, Amparo Alfonso, Carmen Vale, Jorge R. Virués-Segovia, Augustine Mungkaje, Luis M. Botana, Olivier P. Thomas

**Affiliations:** † School of Biological and Chemical Sciences, Ryan Institute, 8799 University of Galway, H91TK33 Galway, Ireland; ‡ Pharmacology Department, Facultad de Veterinaria, 16780 Universidad de Santiago de Compostela, Avenida Carballo Calero s/n, 27002 Lugo, Spain; § Departamento de Química Orgánica, Facultad de Ciencias, Universidad de Cádiz, Cádiz, 11001, Spain; ∥ Instituto de Investigación en Biomoléculas (INBIO), Universidad de Cádiz, Cádiz, 11001, Spain; ⊥ Biological Sciences Discipline, University of Papua New Guinea, P.O. Box 320, University 134, National Capital District, Port Moresby, 9999, Papua New Guinea

## Abstract

Sponges of the genus *Neopetrosia* are known for the production of a large diversity of bioactive metabolites. Contignasterines A (**1**) and B (**2**) were isolated as major metabolites of the sponge *Neopetrosia* cf. *rava* collected in the Bismarck Sea along with the known and highly bioactive steroid contignasterol (**3**) possessing a similar oxidized aglycone. Contignasterines are characterized by the presence of a 2-aminoimidazole branched on the side-chains of the oxidized steroid, and **1** also contains an unusual phosphate group at C-7. The anti-inflammatory activities of these compounds were investigated and revealed that all three compounds inhibited the production of pro-inflammatory mediators.

## Graphical abstract

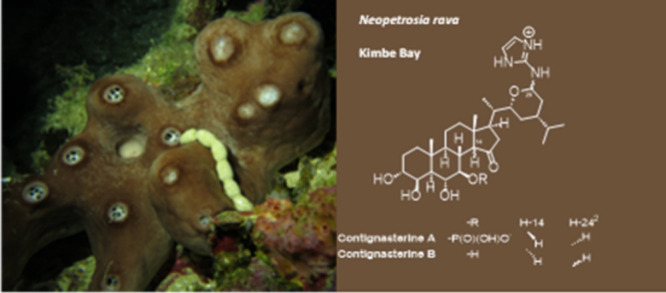

The Coral Triangle located in the western Pacific Ocean is well-known for its extremely rich marine biodiversity.^1^ The intense competition for space within these highly diverse marine ecosystems has likely driven the evolution of a range of specialized metabolites which can be used by marine organism for defense, communication, and reproduction, among other key functions. These ecologically important secondary compounds also form the basis for a range of industrial applications.^2^ As such, the Coral Triangle has historically been and remains a hotspot for marine biodiscovery research.

Sponges are one of the key benthic organisms in coral reef ecosystems competing with other benthic species.^3^ Over the past few decades, studies on several common species of sponges have led to the discovery of important biomolecules with potential for drug discovery.^4^ The *Tara* Pacific expedition was organized between 2016 and 2018 to study the health of coral reefs across the Pacific Ocean. The lack of a systematic inventory of sponges from the Bismarck Sea offered a unique opportunity to collect and study sponges in the region including many understudied groups.^2,5,6^ A set of criteria developed to identify the most promising sponge species based on both biological and chemical diversity led to our first report of novel compounds from sponges in the region.^7^

In the present study, a specimen of a massive dark sponge of the genus *Neopetrosia* was selected for in-depth biological and chemical studies. Based on morphological characters, the sponge was identified as *Neopetrosia* cf. *rava* (Previously *Petrosia rava*).^8^ Its organic extract exhibited a rich LC-MS chemical profile with unknown *m*/*z* values when compared with known marine natural products.^9^ The genus *Neopetrosia* has been recognized as a prolific group of natural products with broad biological activities.^10^ *Neopetrosia proxima* is well-known to produce bioactive 3-alkylpyridinium derivatives while *N. exigua* now named *N. chaliniformis* and *N. compacta* were shown to produce quinone derivatives. This chemical diversity might be related to the difficulty in the identification of species of this group and the polyphyly of the genus *Neopetrosia*.

We report herein the first chemical investigation of the sponge *N.* cf. *rava* (previously known as *Petrosia rava*) collected in North East Papua New Guinea (Kimbe Bay, New Britain) during the *Tara* Pacific expedition. Two novel polyoxygenated steroids (**1** and **2**) bearing an unusual 2-aminoimidazole group on the side-chain, with one of them being phosphorylated at 7-*O*, were isolated from this sponge together with the known contignasterol (**3**), isolated for the first time in 1992 from the sponge *Petrosia contignata* (now *Neopetrosia contignata*).^11,12^ Previous biological assays carried out with contignasterol (**3**) showed its potential to treat asthma,^13^ together with inflammatory and cardiovascular diseases. We report here additional data on the anti-inflammatory potential of this family of steroids.^14–16^ 
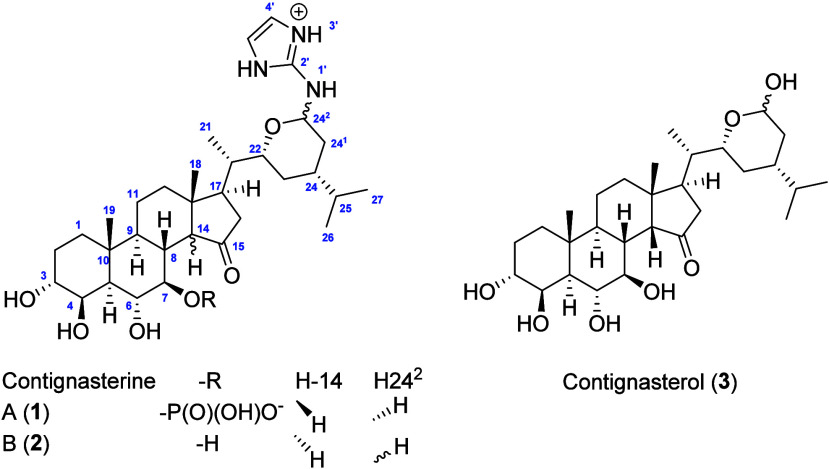


Compound **1** was isolated as a white amorphous solid, whose (+)-HRESIMS spectra showed a protonated molecule [M + H]^+^ at *m*/*z* 654.3524 corresponding to the molecular formula C_32_H_53_N_3_O_9_P. The ^1^H NMR spectrum recorded in methanol-*d*_4_ revealed five methyl signals characteristic of a steroid moiety at *δ*_*H*_ 0.92 (d, *J* = 6.3 Hz, H_3_-26), 0.94 (d, *J* = 6.3 Hz, H_3_-27), 0.96 (d, *J* = 6.7 Hz, H_3_-21), 1.02 (s, H_3_-19), and 1.07 (s, H_3_-18) (Table 1). Oxygenated methines at *δ*_*H*_ 3.82 (br s, H-3), 3.84 (dd, *J* = 11.5, 8.5 Hz, H-6), 4.10 (br s, H-4), and 4.95 (dd, *J* = 15.5, 8.5 Hz, H-7) were reminiscent of oxidations of the steroid backbone. The structure elucidation of the core of the steroid was initiated by using the signals of the methyl groups and their HMBC correlations (Figure 1). Ring A was assigned from HMBC correlations from H_3_-19 to C-1/C-5/C-9/C-10 and the H-1 to H-9 COSY coupled spin system. Two hydroxyl groups were located at H-3 and H-4 due to the chemical shifts of their corresponding signals, and their spin system was extended to H-6, H-7, H-8, and H-9 using COSY correlations and the key H_3_-19/C-9 HMBC correlation. In ring B, two positions C-6 and C-7 were also substituted by an oxygen with the chemical shift at δ_H_ 4.95 (dd, *J* = 15.5, 8.5 Hz, H-7) being too deshielded to correspond to a hydroxyl group. In agreement with the molecular formula a phosphate group was proposed to be located at C-7. The remaining component of the tetracyclic core of the steroid was identified through the COSY correlations and key HMBC correlations from H_3_-18. Similarly to contignasterol (**3**) with oxygenated methines at the same positions in ring A and B,^11^ compound **1** was characterized by a ketone at C-15 of the D ring as revealed by the chemical shift at δ_C_ 222.5 (C-15) and the key H-14/C-15 HMBC correlation. The side chain was identified through key HMBC correlations from H-21, in particular, to an oxygenated methine at δ_C_ 79.5 (C-22) and δ_H_ 3.24 (t, *J* = 9.5 Hz, H-22). This spin system was terminated with an isopropyl group located at C-24 and an unusual deshielded methine at δ_H_ 4.64 (d, *J* = 10.5 Hz, H-29). The steroid skeleton could correspond to a poriferastane or stigmastane, depending on the configuration at C-24. The ^1^H NMR signal of H-24^2^ showed two insightful HMBC correlations: one with C-22 leading to a tetrahydropyrane ring, and another with a nonprotonated and unsaturated carbon at δ_C_ 148.9 (C-2’). The chemical shifts of the signals at C-24^2^ could not correspond to the highly deshielded hemiacetal present in contignasterol (**1**) but rather suggest a hemiaminal. The molecular formula and the chemical shift of C-2’ suggests a guanidine substituted at C-24^2^. The last aromatic methine at δ_C_ 115.3 (C-4’) and δ_H_ 6.71 (s, 2H, H-4’) was linked to the guanidine through the key H-4’/C-2’ HMBC correlation which was in agreement with a 2-aminoimidazole for the substituent at C-24^2^. The 2-aminoimidazole moiety was further supported by the loss of 83 Da observed by HRMS/MS. An additional loss of 95 Da by MS/MS confirmed the presence of the phosphate substituent at C-7. In order to ascertain these unusual features, a second set of NMR experiments were performed in DMSO-*d*_6_ where the signals of the exchangeable protons could be used. A key NH-1’/H-24^2^ COSY unequivocally placed the NH-1’ of the 2-aminoimidazole ring at C-24^2^.^14^ In this solvent, key H-7/C-14, H-7/C-6, and H-7/C-8 HMBC correlations confirmed that the *O*-phosphate group was connected at C-7.

**Table 1:** NMR data for steroids **1** and **2** in methanol-*d*_4_ (^1^H 600 MHz and ^13^C 150 MHz)

			Contignasterine B (**2**) major 1.0	
	Contignasterine A (**1**)		(+minor 0.8)	
Position	*δ*_*C*_, type	*δ*_*H*_, mult. (*J* in Hz)	*δ*_*C*_, type	*δ*_*H*_, mult. (*J* in Hz)
1a	33.1, CH_2_	1.37, m	33.0, CH_2_	1.41, m
1b		1.27, m		1.34, m
2a	24.4, CH_2_	1.93, m	24.3, CH_2_	1.98, m
2b		1.48, m		1.52, m
3	70.6, CH	3.82, br s	70.4, CH	3.83, br s
4	69.9, CH	4.10, br s	69.3, CH	4.05, br s
5	46.4, CH	1.38, m	46.7, CH	1.38, dd (12.0, 3.0)
6	72.4, CH	3.84, dd (11.5, 8.5)	71.2, CH	3.72, dd (12.0, 8.5)
7	80.5, CH	4.95, dd (15.5, 8.5)	79.9, CH	3.29, br t (9.5)
8	39.7, CH	1.63, m	40.8, CH	1.75, q (9.5)
9	48.6, CH	0.90, m	53.5, CH	0.88, m
10	36.3, C	-	36.4, C	-
11a	22.2, CH_2_	1.44, m	21.0, CH_2_	1.65, br d (14.0)
11b		1.15, m		1.30, m
12a	38.3, CH_2_	1.28, m	40.6, CH_2_	2.14, br d (12.5)
12b		1.15, m		1.46, m
13	43.3, C	-	44.8, C	-
14	52.4, CH	2.95, br s	67.9, CH	2.26, d (10.0)
15	222.5, C	-	221.6, C	-
16a	39.7, CH_2_	2.25, dd (19.5)	41.7, CH_2_	2.55, dd (19.0, 8.5)
16b		2.15, d (19.5, 9.5)		2.15, d (19.0)
17	49.8, CH	1.67, m	49.1, CH	1.83, q (10.0)
18	19.6, CH_3_	1.07, s	13.4, CH_3_	0.84, s
19	15.4, CH_3_	1.02, s	15.3, CH_3_	1.05, s
20	39.0, CH	2.03, m	40.3, CH	1.92, m
21	20.6, CH_3_	0.96, d (6.7)	14.2, CH_3_	1.02, d (6.5)
22	79.5, CH	3.24, t (9.5)	78.3, CH	3.50, m
23a	34.5, CH_2_	1.72, br d (12.0)	27.8, CH_2_	1.57, m
23b		0.99, q (12.0)		1.04, m
24	42.8, CH	1.46, m	42.1, CH	1.48, m
25	33.8, CH	1.51, m	33.5, CH	1.54, m
26	19.9, CH_3_	0.92, d (6.3)	19.5, CH_3_	0.95, d (7.0)
27	20.1, CH_3_	0.94, d (6.3)	19.7, CH_3_	0.96, d (7.0)
24^1^a	35.8, CH_2_	1.83, br d (12.0)	34.9, CH_2_	1.94, br d (12.0)
24^1^b		1.33, q (12.0)		1.21, q (12.0)
24^2^	84.4, CH	4.64, d (10.5)	82.8, CH	4.74, dd (10.5, 2.0)
1’	-	-	-	-
2’	148.9, C	-	147.2, C	-
			(148.6, C)	
3′	-	-	-	-
4’	115.3, CH	6.71, s	114.7, CH	6.92, s
			(114.1, CH)	(6.78, s)

**Figure 1:**
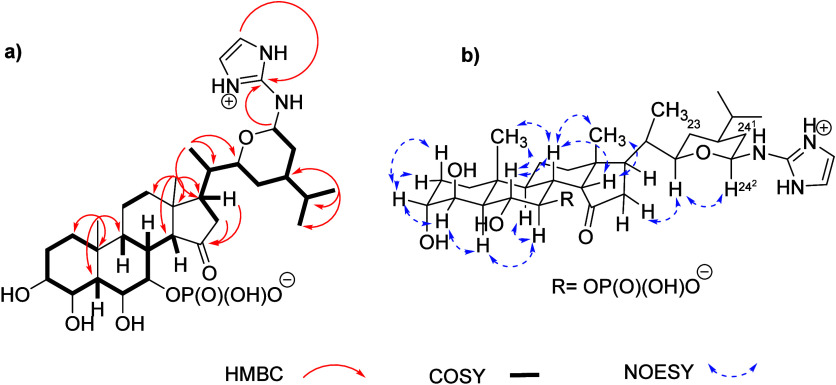
(a) Key HMBC and COSY NMR correlations of **1** in methanol-*d*_4_. (b) Key NOESY NMR correlations of **1** in methanol-*d*_4_.

The relative configuration of **1** was first inferred by comparison with the known and newly isolated contignasterol (**3**). However, the relative and absolute configurations of contignasterol (**3**) have been a subject of debate, especially around the side-chain. The identification of **3** as contignasterol was confirmed by comparison of NMR data run in DMSO-*d*_6_ with those reported in its original description in 1992.^11^ The relative configuration of the ABCD rings in **1** was found to be identical with the one initially found for contignasterol. All coupling constants and nOes were found to be the same as those described for contignasterol. Therefore, H-3 and H-4 are placed in *trans* diequatorial positions as they appear as singlets while H-6 and H-7 are located in *trans* diaxial positions due to a coupling constant of 8.5 Hz between them. The other configurations at C-5/C-8/C-9/C-10/C-13/C-17 were unambiguously assigned through nOes, as indicated in Figure 1b. The configuration at C-14 in the α position of the carbonyl group was found to vary among steroids such as the pandarosides^17,18^ and therefore needed additional assessment. In addition to a clear H_3_-18/H-14 NOE, the chemical shift of C-14 at δ_C_ 52.4 was indicative of the unusual beta orientation for H-14 leading to the rare C/D *cis* junction also shown for contignasterol (**3**) and pandarosides. The electronic circular dichroism (ECD) spectrum of **1** revealed a negative Cotton effect at 302 nm attributed to the n to π* transition of the C-15 ketone, in agreement with a *cis* configuration of C/D ring junction.^19^ For the side-chain, the very clear quartets with *J* = 12 Hz observed for H-23b and H-24^1^b were consistent with a chair conformation and *cis* relative configurations between the three substituents of the tetrahydropyran ring (Figure 1b). For the absolute configuration of the side-chain, inconsistent results were obtained using synthetic approaches. The 22*S*, 24*S* absolute configuration^19^ was corrected as 22*R*, 24*R* a year later.^12^ Due to similar NOE in the conformation shown in Figure 1b, we confirm the configuration as 22*R*, 24*R*. Similar to pandarosides and contignasterol (**3**), **1** is a member of the poriferastane family of steroids found mainly in sponges with an additional activation of the position C-24^2^. Finally, the experimental ECD spectrum of **1** (Figure S4) showed an excellent match with the TD-DFT calculated ECD spectrum of the enantiomer drawn.

Compound **2** was isolated as a white amorphous solid, with a (+)-HRESIMS spectrum showing a protonated molecule [M + H]^+^ at *m*/*z* 574.3859 corresponding to the molecular formula C_32_H_52_N_3_O_6_. Detailed analysis of 2D NMR data of **2** showed key HMBC and COSY correlations similar to those of **1**. In addition, the mass difference of 80 amu between **2** and **1** could indicate replacement of the phosphate group at C-7 in **1** by a hydroxyl group. The ^1^H NMR spectrum of **2** recorded in methanol-*d*_4_ was in accordance with this change, in particular, with the shielding of the signal at *δ*_*H*_ 3.29 (H-7). Importantly, changes were also observed for the NMR signals at C-14. The deshielding of the signal at δ_C_ 67.9 (C-14) and the doublet for H-14 in the ^1^H NMR spectrum replacing the broad singlet in **1** were both in accordance with a change of configuration. H-14 is located in the alpha face of the steroid that corresponds to the more usual *trans* C–D ring junction. This change of configuration was also confirmed by the ECD spectrum of **2** (Figure S16), which revealed a positive Cotton effect at 289 nm attributed to the n to π* transition of the C-15 ketone, in agreement with the 14α configuration.^20^ Epimers at C-14 with a ketone at C-15 were previously isolated from a specimen of *Clathria gombawuaiensis* collected in Gageo-do, Korea,^20^ and from an Oceanapiid sponge collected in Barge Reef, Federated Stated of Micronesia.^21^ The presence of two aromatic signals at δ_H_ 6.92 (s, 1H, H-4’ major) and δ_H_ 6.78 (s, 0.8H, H-4’ minor) in **2** was intriguing as only one was observed for **1** due to the symmetry of the 2-aminoimidazole. The difference in integration between these two singlets led us to consider the presence of rotamers or epimers in the molecule. Considering the known presence of two epimers at C-24^2^ for contignasterol, we hypthesized that compound **2** exists in the form of two epimers at C-24^2^ in a ratio 1:0.8. Indeed, only the NMR signals close to the position C-24^2^ changed between the two chemical entities, and no clear hindered rotation could be proposed for **2**. While **1** is a zwitterion due to the presence of the phosphate, **2** is a cation, and internal ionic interactions between the 2-aminoimidazole and the phosphate are only possible for **1**. This major difference could explain the presence of a preferred epimer for **1** and a mixture of epimers for **2** and **3**.

Compound **3** was isolated as a white amorphous solid, in which the (+)-HRESIMS spectrum showed a sodium adduct ion [M + Na]^+^ at *m*/*z* 531.3289 compatible with a molecular formula C_29_H_48_O_7_Na. NMR data recorded in DMSO-*d*_6_ suggest a mixture of two epimers with similar signals at different integration values. In addition, the ^13^C NMR spectrum of **3** revealed two hemiacetal carbons at *δ*_*C*_ 95.4 and *δ*_*C*_ 90.1 (C-24^2^). These data allowed us to propose that compound **3** corresponds to contignasterol, a compound previously isolated from the sponge *Neopetrosia contignata*.^12,14,15^ Hence, the mixture of epimers in **3** is consistent with the spontaneous epimerization at C-24^2^ originally reported for contignasterol. In this study, it was confirmed by intense ROESY correlations between the axially oriented H-24^2^ (*δ*_*H*_ 4.47) with H-22 (*δ*_*H*_ 3.23) and H-24 (*δ*_*H*_ 1.31) for the 24^2^ *S*-epimer and the absence of a ROESY correlation between equatorially oriented H-24^2^ (*δ*_*H*_ 5.16) with H-22 (*δ*_*H*_ 3.79) and H-24 (*δ*_*H*_ 1.59) for the 24^2^ *R*-epimer. The ECD spectrum of **3** displayed a negative Cotton effect at 312 nm similar to the one for **1** (Figure S27) corresponding to a *cis* configuration for the C/D ring junction.

The biological activity of the compounds was evaluated in the murine macrophage cell line RAW 264.7 to assess their anti-inflammatory potential since contignasterol (**3**) has been shown to exhibit antiasthmatic and anti-inflammatory activity.^22^ Their effects on cell viability and cytotoxicity were determined using the MTT assay and by quantifying the release of lactate dehydrogenase (LDH) into the culture media, respectively. After 24 h of treatment, cell viability reduced by over 50% (53.0 ± 8.1%) in the presence of compound **1** at the highest concentration assayed (10 μM), while none of them increased the release of LDH (Figure 2).

**Figure 2:**
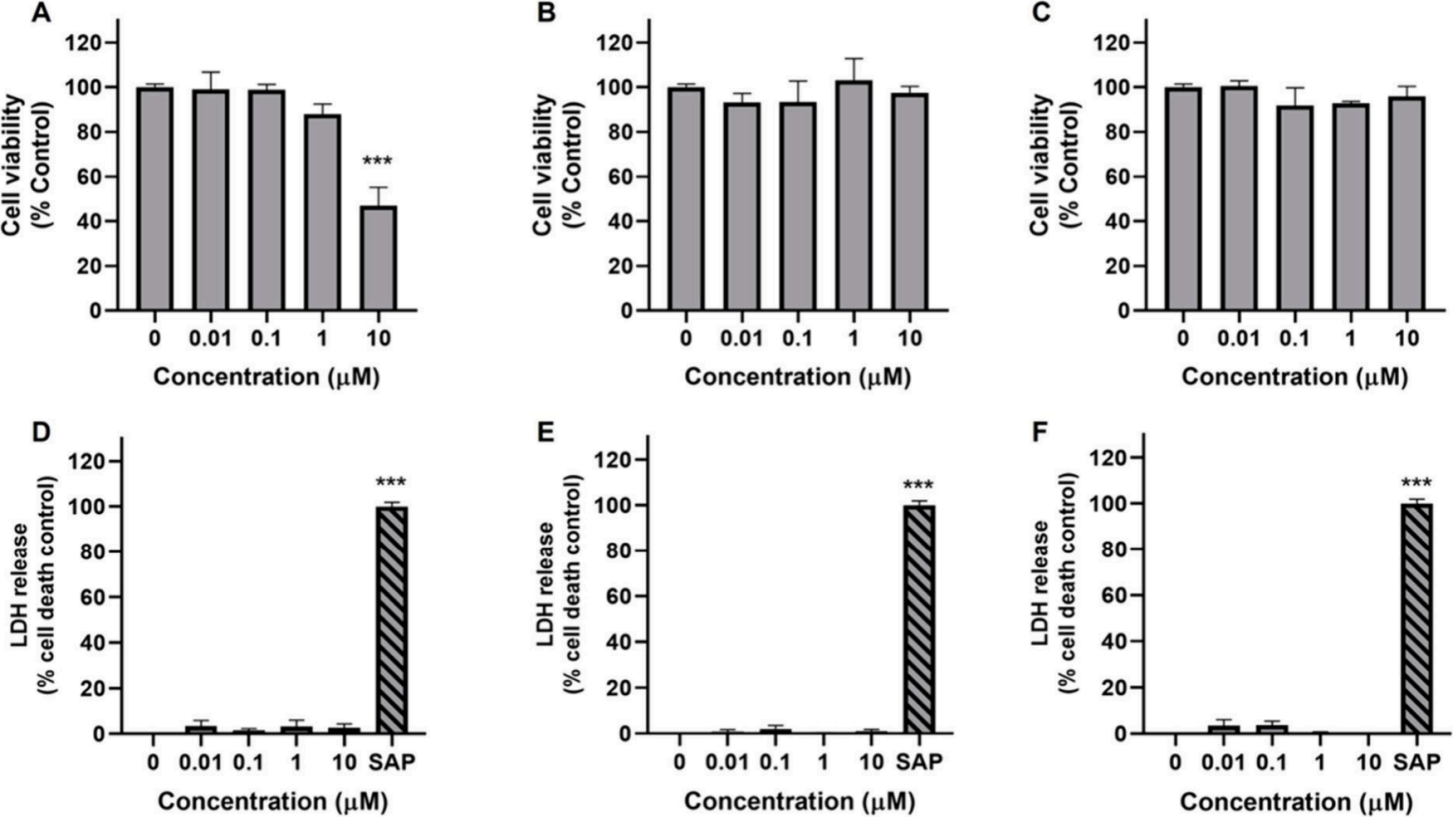
Effect of Contignasterines A and B and Contignasterol on the cell viability and cytotoxicity of RAW 264.7 cells. Cells were treated with the compounds for 24 h. Effects of (A) Contignasterine A (**1**), (B) Contignasterine B (**2**), and (C) Contignasterol (**3**) on cell viability were determined by MTT test. Cytotoxicity of (D) Contignasterine A, (E) Contignasterine B, and (F) Contignasterol assessed by the LDH assay. Saponin (SAP), 1 mg/mL, was used as death control. Mean ± SEM of three independent experiments. Statistical differences determined by One-way ANOVA and Dunnett’s multiple comparison test. ***p ≤ 0.001, significantly different from the control.

This suggests there are nontoxic compounds at concentrations up to 1 μM. Their effect over the release of pro-inflammatory factors was assessed in an inflammatory model in murine macrophages. RAW 264.7 cells were pretreated with different concentrations of compounds (0.001, 0.01, 0.1, and 1 μM) for 1 h, followed by the addition of lipopolysaccharide (LPS) at 1 μg/mL for 24 h. LPS is a component of the cell wall of Gram-negative bacteria that induces inflammation in several immune cell lines, including macrophages.^23^ Activation of macrophages leads to the release of pro-inflammatory mediators such as NO and reactive oxygen species (ROS).^24^ None of the compounds induced an increase in NO production when macrophages were treated with them alone, confirming the lack of pro-inflammatory properties of these compounds (Figure 3).

**Figure 3:**
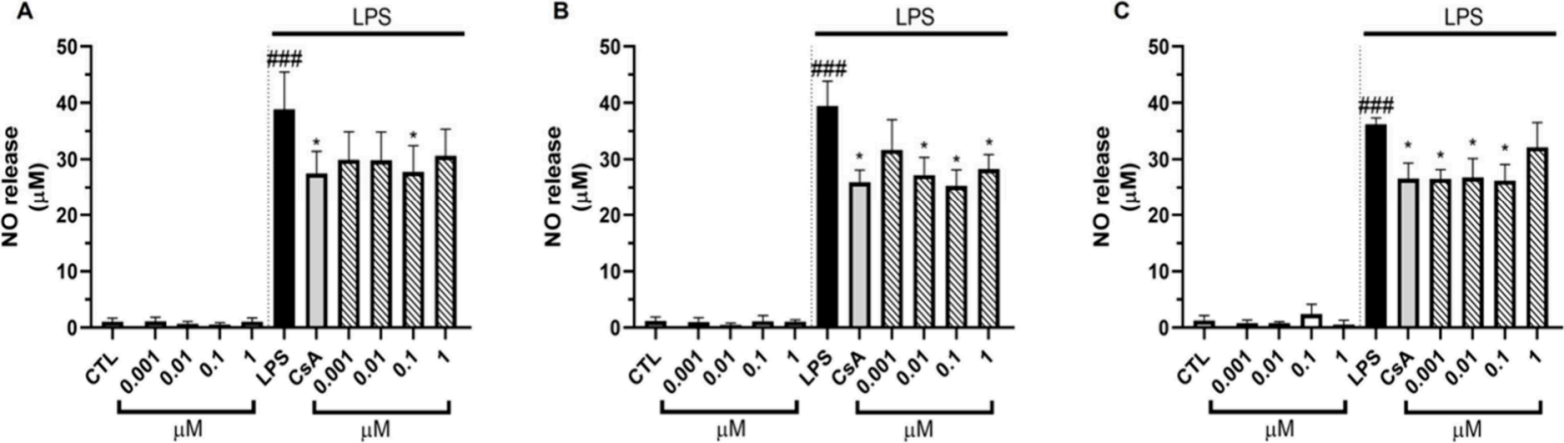
Effect of Contignasterines A and B and Contignasterol on NO release in murine macrophages. Cells were pretreated with (A) Contignasterine A, (B) Contignasterine B, and (C) Contignasterol for 1 h and activated with 1 μg/mL LPS for 24 h. Cyclosporine A (CsA) at 1 μM was used as anti-inflammatory control. Data are mean ± SEM of three independent experiments. Statistical significance determined by One-way ANOVA and Dunnett’s multiple comparison test. *p ≤ 0.05, compared to LPS treated cells; ###*p* ≤ 0.001, compared to control cells.

In addition, the three compounds were able to inhibit the release of NO induced by LPS in a similar way to cyclosporin A (CsA), a well-known anti-inflammatory drug, with compounds **2** and **3** being most active. NO release was reduced in the presence of compound **2** at concentrations ranging from 0.01 to 1 μM (Figure 3B), while compound **3** was active among 0.001 to 0.1 μM (Figure 3C). The next step was to investigate whether these compounds modulate the production of ROS. As shown in Figure 4, the treatment with LPS increased ROS production up to 160%, while the preincubation with CsA was able to almost completely prevent the production of these molecules.

**Figure 4:**
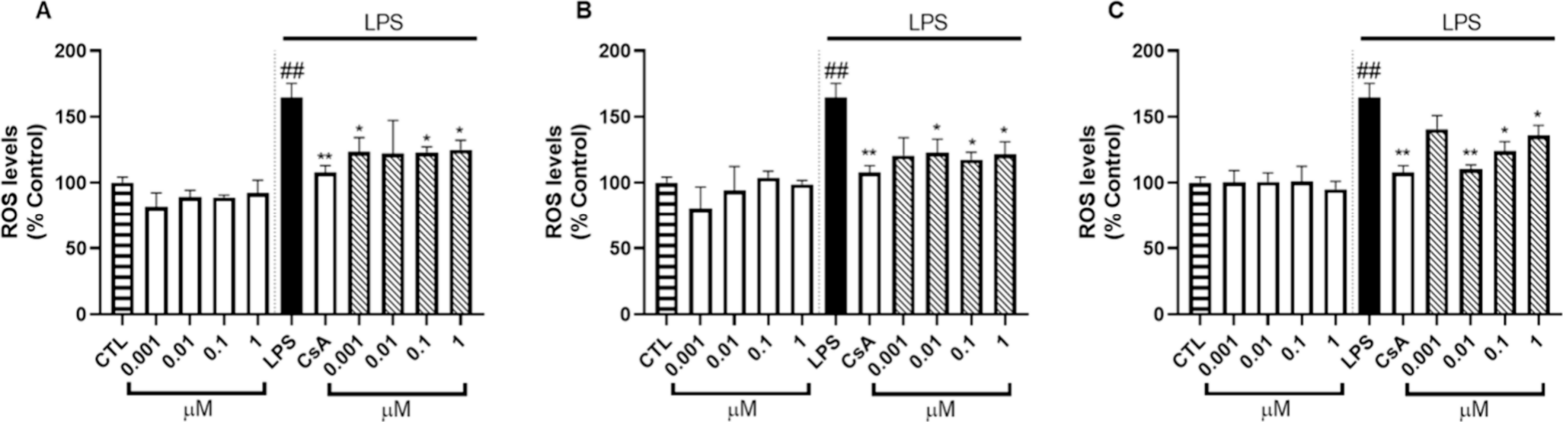
ROS levels modulation by compounds in RAW 264.7 macrophages. Cells were pretreated for 1 h with (A) Contignasterine A, (B) Contignasterine B, or (C) Contignasterol, followed by activation with 1 μg/mL LPS for 24 h. Cyclosporine A (CsA) at 1 μM was used as anti-inflammatory control. Data expressed as mean ± SEM of three independent experiments. Statistical significance was determined by One-way ANOVA and Dunnett’s multiple comparison test. *p ≤ 0.05, **p ≤ 0.01 compared to inflammatory control cells; ##*p* ≤ 0.01, compared to control cells.

The same effects were observed when cells were pretreated with contignasterines or contignasterol. ROS was significantly reduced at levels as low as those observed in the control without LPS treatment. Compounds **1** and **2** were again the most active, reinforcing their role as potential anti-inflammatory agents. Finally, in order to clarify the mode of action of these compounds, their effect over the transient receptor potential vanilloid (TRPV1) or vanilloid receptor subtype 1 channels was studied. These calcium channels are associated with several pathologic and physiologic processes, including inflammation. TRPV1 is activated by capsaicin, the endogenous agonist anandamine, low pH, high temperature, or voltage.^25^ The effect of the three compounds was tested by electrophysiological measurements in HEK293 cells expressing human TRPV1 channels. No effects over TRPV1 channels were observed with contignasterines, but contignasterol did block 50% of the channel response at 5 μM, and 20 μM elicits a 75% inhibition (Figure 5).

**Figure 5:**
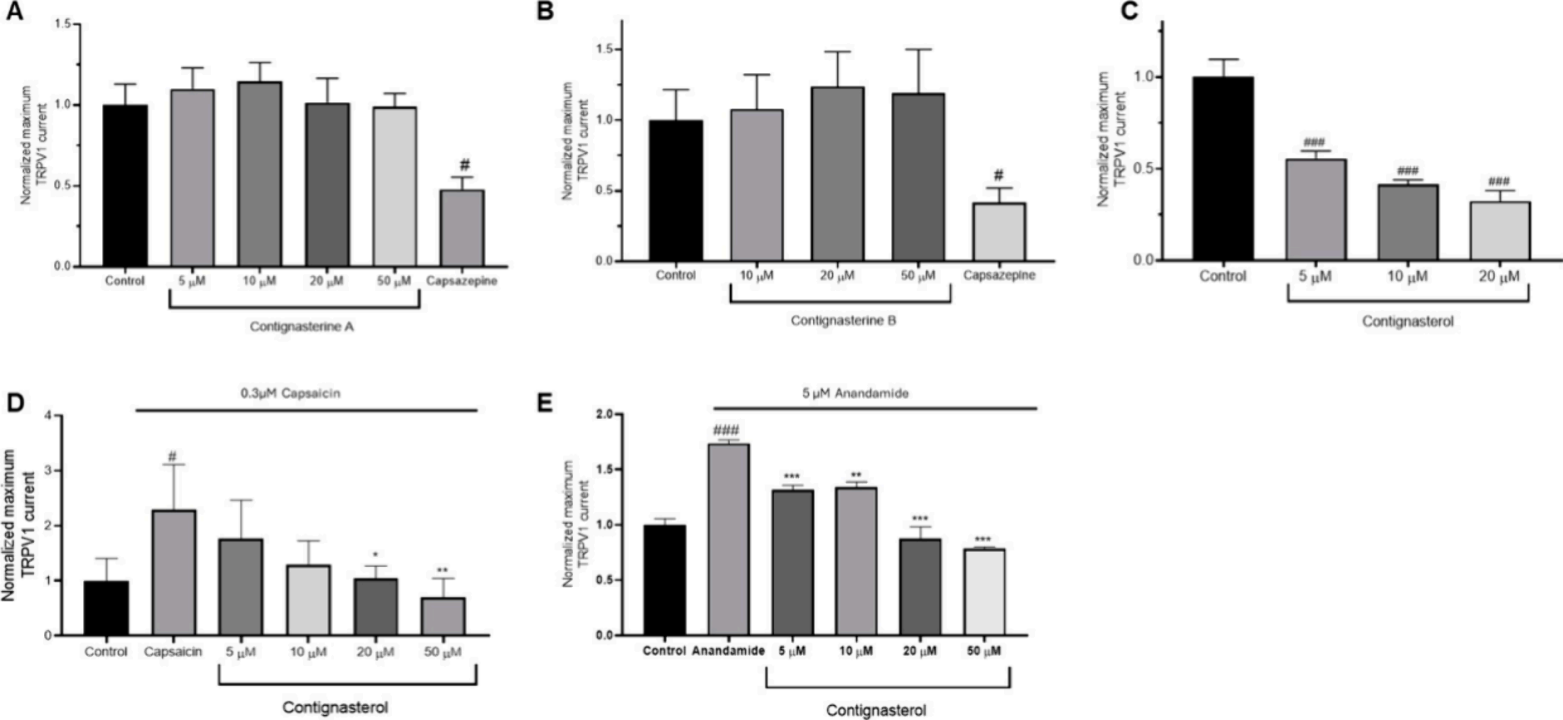
Effect of compounds on TRPV1 currents amplitude. Averaged maximum TRPV1 current intensity at 100 mV after bath application of (A) Contignasterine A, (B) Contignasterine B, or (C) Contignasterol. Capsazepine, 50 μM, was used to antagonize TRPV1 currents. Averaged maximum TRPV1 current intensity at 100 mV after bath application of capsaicin, 0.3 μM (D) or anandamide, 5 μM (E), followed by contignasterol treatment. Data expressed as mean ± SEM. Statistical significance determined by One-way ANOVA and Dunnett’s multiple comparison test. #*p* ≤ 0.05, ###*p* ≤ 0.001 compared to control cells; *p ≤ 0.05, **p ≤ 0.01, ***p ≤ 0.001 compared to TRPV1 agonists.

Since these channels are operated by ligands, 0.3 μM capsaicin and 5 μM anandamide were used to open the channel. Figure 5D,E shows that the effect of these ligands was inhibited by 100% with 50 μM contignasterol. This inhibitory response suggests that contignasterol is a multitarget molecule that may block the channels, compete with the ligands, or allosterically modulate their binding. Inflammation is a critical component of many physiological processes, aiding in defense and tissue repair. However, an uncontrolled inflammatory response can lead to chronic inflammation and significant tissue damage. This exaggerated response is a driving factor in many diseases, highlighting the importance of effectively managing inflammation.^26^ Therefore, compounds that modulate inflammation are promising drug candidates for the development of new therapies. Contignasterines and contignasterol inhibit ROS production and NO release in macrophages, making them promising lead compounds. In addition, contignasterol also affects TRPV1 channels, suggesting that it may show an anti-inflammatory effect through several mechanisms. On the other hand, the potential anti-inflammatory effect of contignasterines is not mediated through TRPV1 channels, as they target only mechanisms associated with redox species.

## Conclusions

We present here the second report on the isolation of contignasterol from a sponge collected from the Bismarck Sea. The morphological analyses of the sample collected in this study was inconsistent with the species *Neopetrosia contignata* in which contignasterol (**3**) was previously identified from.^11^ Sponges are notoriously difficult to identify based on morphology alone, and while this specimen might be similar to *N. contignata* in the production of contignasterol, it matched more closely to *N. rava* based on morphology.^8^ *Neopetrosia rava* is only known from its type locality in the Sulawesi Sea, which is in close proximity to Papua New Guinea.^27^ Neither species has available barcodes for comparison and may represent closely related species. Alternatively, the compound may be produced by an associated microorganism shared by different sponge species in this region. Interestingly, we report for the first time the inclusion of a 2-aminoimidazole moiety on the side chain of contignasterines. Similar oxidized but sulfated steroids were isolated from a sponge of the genus *Xestospongia* later reassigned to *Cribrochalina*, both in the order Haplosclerida. Here again it shows how the taxonomy of this group is highly challenging.^28^ Interestingly, these two metabolites named haplosomates were found later in a sponge of the genus *Cribrochalina*, and their structures were revised as phosphorylated and not sulfated oxidized steroids later.^29^ The presence of a phosphate group in compound **1** is also unusual, and its zwitterionic nature may account for a specific conformation that prevents the formation of the C-24^2^ epimer observed in compounds **2** and **3**. Contignasterol and its new derivative contignasterines belong to the class poriferastane, which are unique steroids found in sponges with a position C-24^2^ oxidized into an aldehyde leading to the creation of a unique tetrahydropyrane ring on the side chain. Contignasterol (IZP-94005 as developed by the spin-off company Inflazyme Pharmaceuticals) was found to be a good candidate as an antiasthma drug due to its antihistaminic bioactivity, and *in vivo* bioassays were promising.^13^ However, no mechanism of action was evidenced with this compound, and a series of simpler analogues were synthetically produced leading to IPL576,092 being 10-fold more active than the natural product.^22^ Unfortunately, the commercialization of this antiasthma derivative by the spin-off failed in Phase II clinical trials leading the company to cease its activities. The discovery of structural natural analogues of contignasterol in a sponge widely distributed in Kimbe Bay is promising to broaden the scope of this anti-inflammatory agent.

## Experimental Section

### General Experimental Procedures

Optical rotations were recorded at 589 nm (the Na D line) on a Rudolph Research Analytical Autopol IV automatic polarimeter at room temperature. UV and ECD measurements were achieved on a Chirascan V100 spectrophotometer (Applied Photophysics, Leatherhead, UK). 1D and 2D NMR spectra were obtained on an Agilent Premium Compact 600 MHz spectrometer (Agilent, CA, USA) fitted with a 5 mm cryoprobe, using deuterated methanol (MeOH-*d*_4_) or dimethyl sulfoxide (DMSO-*d*_6_) as solvents. Chemical shifts were expressed in parts per million and referenced to the residual solvent signals (MeOH-*d*_4_, at *δ*_*H*_ 3.31 and *δ*_*C*_ 49.00 ppm; DMSO-*d*_6_, at *δ*_*H*_ 2.50 and *δ*_*C*_ 39.52 ppm). High-resolution mass spectra data (HRMS) were acquired by using an Agilent 6540 Q-Tof mass spectrometer UHPLC–DAD–HRMS (Agilent 6540). Purifications were carried out using two instruments: (a) a semipreparative HPLC-UV system equipped with a Jasco PU-2087 pump and UV-2075 detector (Tokyo, Japan); (b) an Agilent 1260 HPLC system equipped with a DAD detector.

### Biological Material

The specimen (MBNUIG 229), identified as the species *Neopetrosia* cf. *rava*, was collected as part of a broader biodiversity assessment of sponges from Kimbe Bay, Bismarck Sea, Papua New Guinea. A voucher specimen is preserved in 99% ethanol in our repository.^2^ For the biological description of the specimen, refer to Supporting Information Figure S1.

### Extraction and Isolation

A sample (55.0 g) of the sponge, previously freeze-dried and ground, was extracted using a mixture of MeOH/H_2_O (1:1, v/v, 3 × 300 mL) in an ultrasonic bath at room temperature. The dried extract (6.2 g) was fractionated by flash silica C-18 vacuum liquid chromatography (VLC) in seven fractions with solvents of decreasing polarity: H_2_O, H_2_O/MeOH (1:1 and 1:3), MeOH, MeOH/CH_2_Cl_2_ (3:1 and 1:1), and CH_2_Cl_2_. The methanolic fraction (0.39 g) was purified by semipreparative HPLC on a C-18 column (Waters XBridge BEH Shield PP18, 5 μm; 10 × 250 mm; flow rate 3.0 mL/min) with CH_3_CN–H_2_O as the mobile phase and using a gradient method: linear gradient from 34% to 36% CH_3_CN in 20 min; linear gradient from 36% to 100% CH_3_CN in 2 min; 100% CH_3_CN for 10 min. As a result, six subfractions (A–F) were obtained. Subfraction D (6.55 mg) was purified by HPLC on a phenyl-hexyl column (Waters Xselect CSH, 5 μm; 4.6 × 250 mm; flow rate 1.0 mL/min) using an isocratic method (CH_3_CN/H_2_O, 75:25) yielding compound **1** (0.91 mg). Compound **2** was identified pure in subfraction A (1.43 mg). Subfraction F (16.4 mg) was purified by HPLC on a C-18 column (Waters Xbridge C18, 5 μm; 4.6 × 250 mm; flow rate 0.8 mL/min) using an isocratic method (CH_3_CN/H_2_O, 50:50) yielding compound contignasterol (**3**) (0.78 mg).

#### Contignasterine A (1)

White amorphous solid; [*a*]_*D*_^20^ + 9.3 (*c* 0.015, MeOH); UV (MeOH) λ_max_ (log ε) 214 (4.00), 285 (2.92); ECD (*c* 7.65 × 10^–5^ M, MeOH) λ_max_ (Δε) 302 (−4.0) nm; (+)-HRESIMS *m*/*z* 654.3524 [M + H]^+^ (calcd for C_32_H_53_N_3_O_9_P^+^, 654.3514; for ^1^H and ^13^C NMR data, see Table 1.

#### Contignasterine B (2)

White amorphous solid; UV (MeOH) λ_max_ (log ε) 208 (4.23), 317 (3.12); ECD (*c* 8.71 × 10^–5^ M, MeOH) λ_max_ (Δε) 289 (+4.2) nm; (+)-HRESIMS *m*/*z* 574.3859 [M + H]^+^ (calcd for C_32_H_52_N_3_O_6_^+^, 574.3851); for ^1^H and ^13^C NMR data, see Table 1.

#### Contignasterol (3)

White amorphous solid; UV (MeOH) λ_max_ (log ε) 282 (2.47); ECD (*c* 1.97 × 10^–4^ M, MeOH) λ_max_ (Δε) 312 (−2.0) nm; (+)-HRESIMS *m*/*z* 531.3289 [M + Na]^+^ (calcd for C_29_H_48_O_7_Na^+^, 531.3298); for ^1^H and ^13^C NMR data, see Table S1.

### Computational Methods

Conformational analysis was performed in Schrodinger MacroModel (v 11.8) using a Monte Carlo Minimum Method and the molecular mechanics OPLS_2005 force field with an energy cutoff of 5 kcal/mol and constraining the conformations using key ROESY correlations. All quantum mechanical calculations were performed using the Gaussian 16 package.^30^ First, an initial geometrical optimization was conducted through the application of the semiempirical PM6 method. Following this a second geometrical optimization and a frequency check was carried out using the density functional theory (DFT) within the framework of the B3LYP functional and the 6-31+G­(d,p) basis set. Subsequently, calculations were performed to determine the energies, oscillator strengths, and rotational strengths associated with the initial 20 electronic excitations, employing the TD-DFT methodology within the framework of the B3LYP functional and the 6-311+G­(2d,p) basis set. The influence of the solvent (methanol) was considered within the calculations, incorporating the polarizable continuum model (PCM) with the implementation of the implicit solvation energy (IEF) approach.^31^ The resulting ECD spectra were obtained after Boltzmann averaging using Specdis 1.7.^32^ Finally, to mimic the ECD spectrum of the conformer, a Gaussian function was used featuring a half-bandwidth of 0.33 eV.

### Biological Assays

#### Chemicals and Solutions

CyQUANT lactate dehydrogenase (LDH) Cytotoxicity Assay Kit, Dulbecco’s Modified Eagle Medium (DMEM), Dulbecco’s Modified Eagle Medium: F-12 nutrient Mix (DMEM/F-12), and supplements used for cell cultures were purchased from Thermo Fisher Scientific (Madrid, Spain), while all other chemical grade reagents were purchased from Merck (Madrid, Spain). The composition of Locke’s buffer was: 154 mM NaCl, 5.6 mM KCl, 3.6 mM NaHCO_3_, 1 mM MgCl_2_ 1.3 mM CaCl_2_, 5 mM glucose, and 10 mM HEPES (pH 7.4). Phosphate buffered saline (PBS) was composed of (in mM): 137.0 NaCl, 8.2 Na_2_HPO_4_, 1.5 KH_2_PO_4_, and 3.2 KCl (pH 7.4).

#### Cell Culture

Murine macrophages (RAW 264.7) were cultured in DMEM medium supplemented with 10% fetal bovine serum (FBS), 10,000 U/mL penicillin-streptomycin, and 1% glutamine. Cells were maintained at 37 °C in a humidified atmosphere of 5% CO_2_ and 95% air and dissociated twice a week. Human embryonic kidney cell line (HEK293) expressing the human TRPV1 channels was cultured in DMEM/F12 medium supplemented with 1% glutamax, 1% nonessential amino acids solution, 10% fetal bovine serum, and 0.4 mg/mL Geneticin and maintained at 37 °C in a humidified 95% O_2_/5% CO_2_ atm, replacing the medium every 2 days. For electrophysiological experiments, cells were subcultured in 12-well plates in glass coverslips at a density of 40,000 cel/mL.

#### Viability and Cytotoxicity Assays

The evaluation of the effects of contignasterines A and B and contignasterol on cell viability and cytotoxicity was performed using the MTT assay and the CyQUANT LDH Cytotoxicity Assay Kit test, respectively, as previously described.^33^

Cells were seeded in 384-well plates at a concentration of 2.5 × 10^4^ cells/well and allowed to grow for 24 h. Treatment with the compounds was then carried out for 24 h at concentrations between 0.01 and 10 μM. Saponin (SAP) from Quillaja bark (1 mg/mL) was used as a cell death control.

LDH release was evaluated by transferring 50 μL of cell culture medium to a 96-well flat-bottom plate. Reagents from the CyQUANT LDH Cytotoxicity Assay Kit were then added according to the manufacturer’s instructions. Absorbance was measured at 490 and 680 nm using a plate reader. The background signal (680 nm absorbance) was subtracted from the 490 nm absorbance to quantify the LDH release into the medium. All experiments were conducted independently three times, each in triplicate.

For the viability assay, cells were washed three times with Locke’s solution. Then, 200 μL of MTT (500 μg/mL) was added per well, and cells were then incubated at 37 °C for 1 h with shaking at 300 rpm on an orbital shaker. After incubation, MTT solution was removed, and 5% sodium dodecyl sulfate was added to lyse the cells. Absorbance was measured by using a plate reader spectrophotometer at 595 nm.

#### NO Determination

The NO concentration in the culture media was analyzed with the Griess method, which detects the formation of nitrite by the spontaneous oxidation of NO, as previously described.^34^ Cells were seeded in 24-well plates (5 × 10^5^ cells per well) in phenol red-free culture medium and preincubated with the compounds at concentrations ranging from 0.001 to 1 μM for 24 h, 1 h prior to stimulation with 1 μg/mL lipopolysaccharide (LPS). Cyclosporine A (CsA), 1 μM, was used as an anti-inflammatory control, and the compounds were tested both alone and in the presence of LPS. Then, 150 μL of culture medium was combined with 50 μL of Griess reagent (1% sulfanilamide in 2.5% phosphoric acid and 0.1% naphthylethylenediamine dihydrochloride). After incubation for 30 min at room temperature in the dark, nitrite levels were quantified by measuring the absorbance at 546 nm using a spectrophotometer plate reader. All measurements were made in triplicate, three independent times.

#### Measurement of Intracellular Reactive Oxygen Species Levels

The fluorescent dye carboxy-H_2_-DCFDA was used to measure the reactive oxygen species **(**ROS) production in macrophages as described before.^35^

Cells were seeded in 384-well plates at a concentration of 2.5 × 10^4^ cells/well and allowed to grow for 24 h. Subsequently, murine macrophages were pretreated with compounds (0.001, 0.01, 0.1, and 1 μM) for 24 h, 1 h before stimulation with LPS (1 μg/mL). The cells were then washed twice with serum-free medium and incubated with 20 μM carboxy-H2-DCFDA for 1 h at 37 °C. The cells were rinsed with PBS and incubated for 30 min at 37 °C. Intracellular ROS production was measured by detecting the fluorescence on a spectrophotometer plate reader (495 nm excitation and 535 nm emission).

#### Electrophysiological Recordings

For whole cell patch-clamp recordings, cells were maintained at room temperature in a recording chamber with 0.5 mL of Locke’s buffer as extracellular solution. The pH was maintained at 7.4 with a Trizma base for electrophysiological experiments. Recording electrodes, fabricated with borosilicate glass microcapillaries (1.5 mm outer diameter), had resistances ranging from 5 to 10 MΩ. Two different pipet solutions were used. One of them contained (in mM): 120 mM CsF, 10 mM EGTA, 10 mM HEPES, and 15 mM NaCl and was adjusted to a pH of 7.25. A second intracellular solution containing ATP was used, since TRPV1 channels are sensitive to intracellular ATP.^36^ Cells were maintained at a holding potential (Vhold) of–55 mV, and 200 ms voltage steps from −100 to +100 mV in 10 mV step increments were applied to record TRPV1 channel activation with a Multiclamp 700B amplifier and digitalized with the Digidata 1440A (both from Axon Instruments, California, U.S.A.). Signals were sampled at 50 kHz after low pass Bessel filtering at 10 kHz and analyzed using pClamp 10 software (Axon Instruments). Series resistance was compensated for by about 70%.

#### Statistical Analysis

All data are expressed as mean ± SEM. Data analysis was performed using GraphPad Prism 8. Statistical comparisons were performed using one-way ANOVA followed by post hoc Dunnett’s tests. *p* values <0.05 were considered statistically significant.

## Supplementary Material





## Notes

Underwater photo of the sample. Biological description of the sponge. NMR, experimental and calculated ECD and MS spectra for compounds **1**–**3** (PDF)
NMR spectra FID files uploaded in NP-MRD under the following numbers: NP0350639, NP0350640, and NP0080544 (ZIP)
