# Supplementary material for: Contignasterines, Anti-Inflammatory 2‑Aminoimidazole Steroids from the Sponge Neopetrosia cf. rava Collected in the Bismarck Sea
Source: J Nat Prod. 2025 Mar 7;88(5):1244–52. doi: 10.1021/acs.jnatprod.5c00118 (PMC12105020; doi:10.1021/acs.jnatprod.5c00118)
Supplement: Supplementary file 1 [file np5c00118_si_001.pdf]

Supplementary material for

Contignasterines, anti-inflammatory 2-aminoimidazole steroids from the sponge *Neopetrosia* cf. *rava* collected in the Bismarck Sea

Juan Ortega-Vidal,[a] Maggie M. Reddy,[a] Nadia Pérez-Fuentes,[b] Rebeca Alvariño,[b] Svenja Burth,[a] Amparo Alfonso,[b] Carmen Vale,[b] Jorge R. Virués-Segovia[c,d], Augustine Mungkaje,[e] Luis M. Botana,[b,] Olivier P. Thomas[a,]**

a School of Biological and Chemical Sciences, Ryan Institute, University of Galway, H91TK33 Galway, Ireland

b Pharmacology Department, Facultad de Veterinaria, Universidad de Santiago de Compostela, Avenida Carballo Calero s/n, 27002 Lugo, Spain

c Departamento de Química Orgánica, Facultad de Ciencias, Universidad de Cádiz, Cádiz, Spain

d Instituto de Investigación en Biomoléculas (INBIO), Universidad de Cádiz, Cádiz, Spain

e Biological Sciences Discipline, University of Papua New Guinea, P.O Box 320, University 134, National Capital District, Port Moresby, Papua New Guinea

List of Figures

Figure S 1. Underwater photo of the sponge *Neopetrosia rava* collected in Kimbe Bay PNG. 3
Figure S 2 Spicule images taken with a Scanning Electron Microscope at 15.0 kV. All spicules are monaxons, mostly with two sharp sides, but sometimes one or both sides are rounded, especially in smaller spicules........ 4
Figure S 3. (+)-HRESIMS analysis of compound 1........ 5
Figure S 4. Comparison of ECD spectra for compound 1 in CH_3OH. 5
Figure S 5. ^{1}H NMR spectrum of 1 (CD_3OD, 600 MHz). 6
Figure S 6. ^{13}C NMR spectrum of 1 (CD_3OD, 150 MHz). 6
Figure S 7. COSY NMR spectrum of 1 (CD_3OD, 600 MHz). 7
Figure S 8. HSQC NMR spectrum of 1 (CD_3OD, 600 MHz)........ 7
Figure S 9. HMBC NMR spectrum of 1 (CD_3OD, 600 MHz)........ 8
Figure S 10. NOESY NMR spectrum of 1 (CD_3OD, 600 MHz). 8
Figure S 11. ^{1}H NMR spectrum of 1 (DMSO-d_6, 600 MHz). 9
Figure S 12. ^{13}C NMR spectrum of 1 (DMSO-d_6, 150 MHz). 9
Figure S 13. COSY NMR spectrum of 1 (DMSO-d_6, 600 MHz)........ 10
Figure S 14. NOESY NMR spectrum of 1 (DMSO-d_6, 600 MHz). 10
Figure S 15. (+)-HRESIMS analysis of compound 2........ 11
Figure S 16. Comparison of ECD spectra for 2 in CH_3OH........ 11
Figure S 17. ^{1}H NMR spectrum of 2 (CD_3OD, 600 MHz). 12
Figure S 18. COSY NMR spectrum of 2 (CD_3OD, 600 MHz). 12
Figure S 19. HSQC NMR spectrum of 2 (CD_3OD, 600 MHz)........ 13
Figure S 20. HMBC NMR spectrum of 2 (CD_3OD, 600 MHz)........ 13
Figure S 21. ^{1}H NMR spectrum of 2 (DMSO-d_6, 600 MHz). 14
Figure S 22. COSY NMR spectrum of 2 (DMSO-d_6, 600 MHz)........ 14
Figure S 23. HSQC NMR spectrum of 2 (DMSO-d_6, 600 MHz)........ 15
Figure S 24. HMBC NMR spectrum of 2 (DMSO-d_6, 600 MHz)........ 15
Figure S 25. NOESY NMR spectrum of 2 (DMSO-d_6, 600 MHz). 16
Figure S 26. (+)-HRESIMS analysis of compound 3........ 17
Figure S 27. Comparison of ECD spectra of 3 in CH_3OH. 17
Table S 1 NMR data for 24^2S and 24^2R epimers of contignasterol (3) in DMSO-d_6 (^{1}H 600 MHz and ^{13}C 150 MHz). 18
Figure S 28. ^{1}H NMR spectrum of 3 (DMSO-d_6, 600 MHz). 19
Figure S 29. COSY NMR spectrum of 3 (DMSO-d_6, 600 MHz)........ 20
Figure S 30. HSQC NMR spectrum of 3 (DMSO-d_6, 600 MHz)........ 20
Figure S 31. HMBC NMR spectrum of 3 (DMSO-d_6, 600 MHz)........ 21
Figure S 32. ROESY NMR spectrum of 3 (DMSO-d_6, 600 MHz)........ 21

Biological material

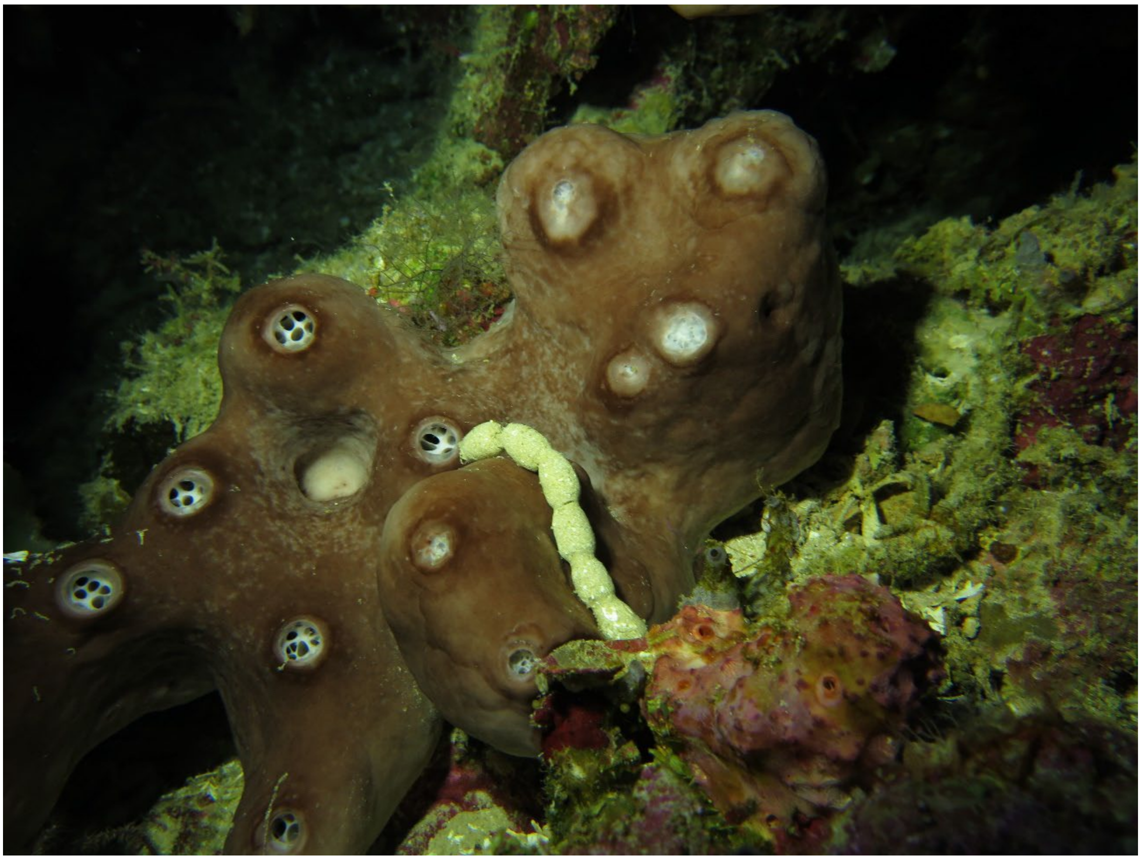

Figure S 1. Underwater photo of the sponge *Neopetrosia* cf. *rava* collected in Kimbe Bay PNG.

Description:

As described by Thiele (1899) the sponge has a brown colour. Its ossicles, which are surrounded by lifted edges, are very big and in each osculum several separate channels develop. The skeleton is fragile and mostly made up of spongin which forms rectangular structures. The spicules are distributed within the spongin structure, following the same organization. The spicules are made up of monaxons of varying sizes (40μm-190μm). Most of them have short sharp points, but some have one or two rounded points, especially the smaller spicules.

Reference:

Thiele, J. (1899). Studien über pazifische Spongien. II. Ueber einige Spongien von Celebes. Zoologica. *Original-Abhandlungen aus dem Gesamtgebiete der Zoologie. Stuttgart.* 24 (2): 1-33, pls I-V.

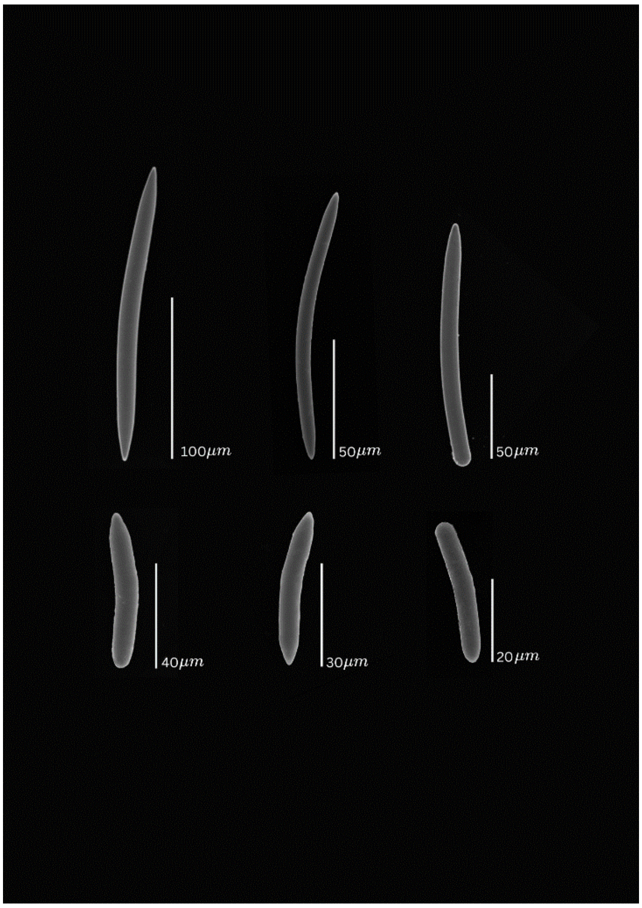

Figure S 2 Spicule images taken with a Scanning Electron Microscope at 15.0 kV. All spicules are monaxons, mostly with two sharp sides, but sometimes one or both sides are rounded, especially in smaller spicules.

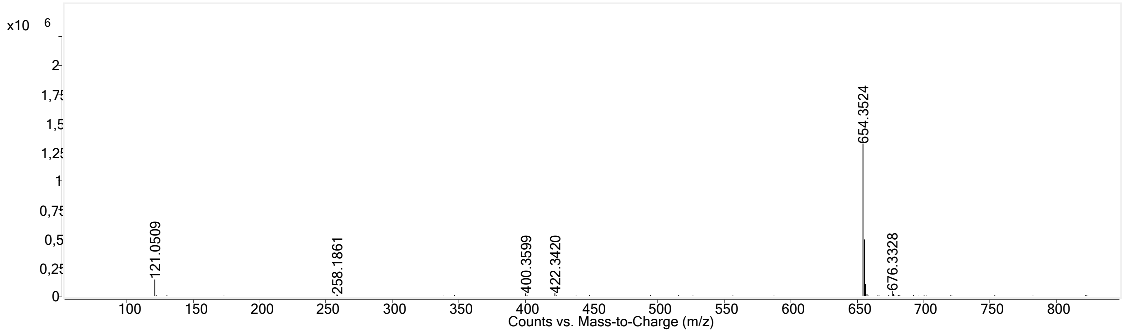

Figure S 3. (+)-HRESIMS analysis of compound **1.**

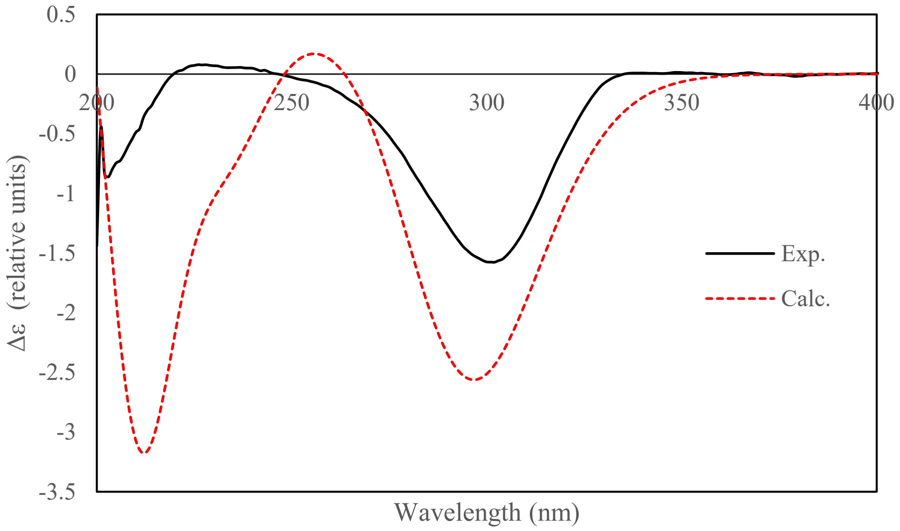

Figure S 4. Comparison of ECD spectra for compound **1** in CH_3OH.

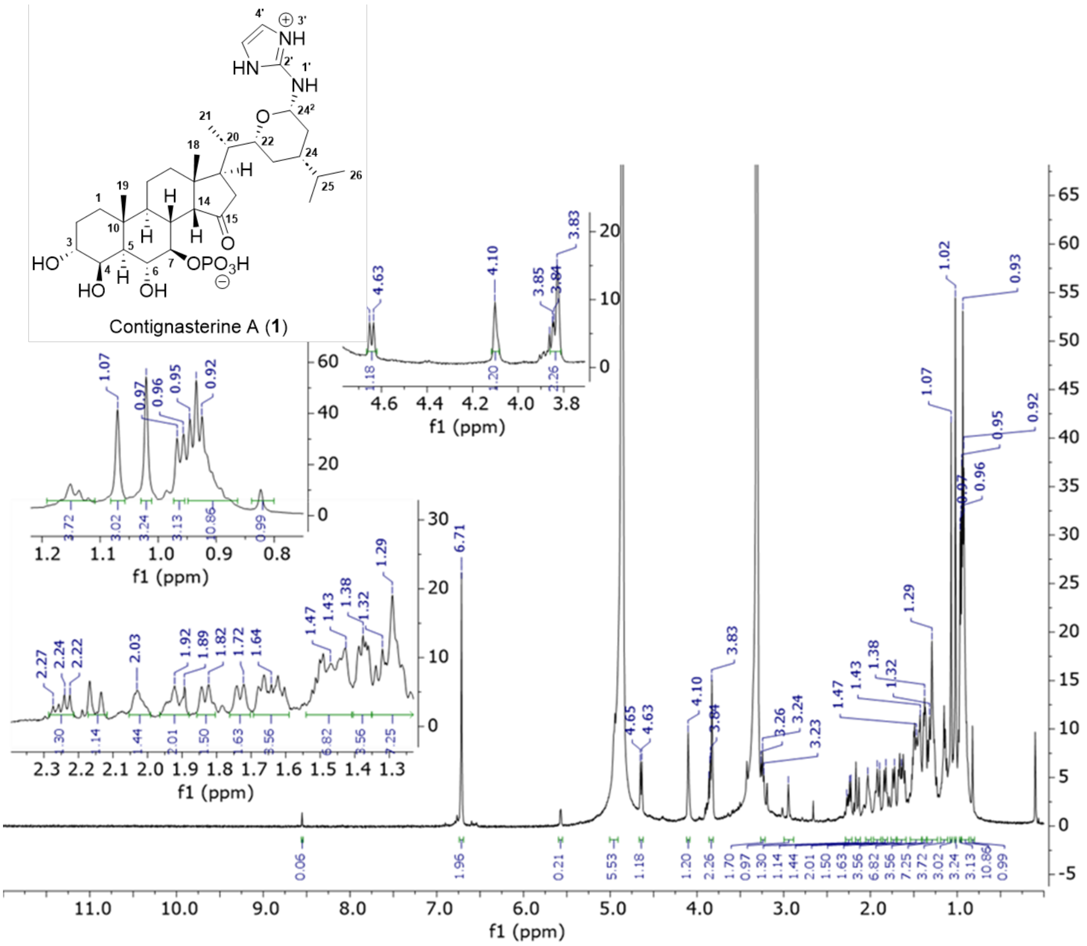

Figure S 5. ^{1}H NMR spectrum of **1** (CD_3OD, 600 MHz).

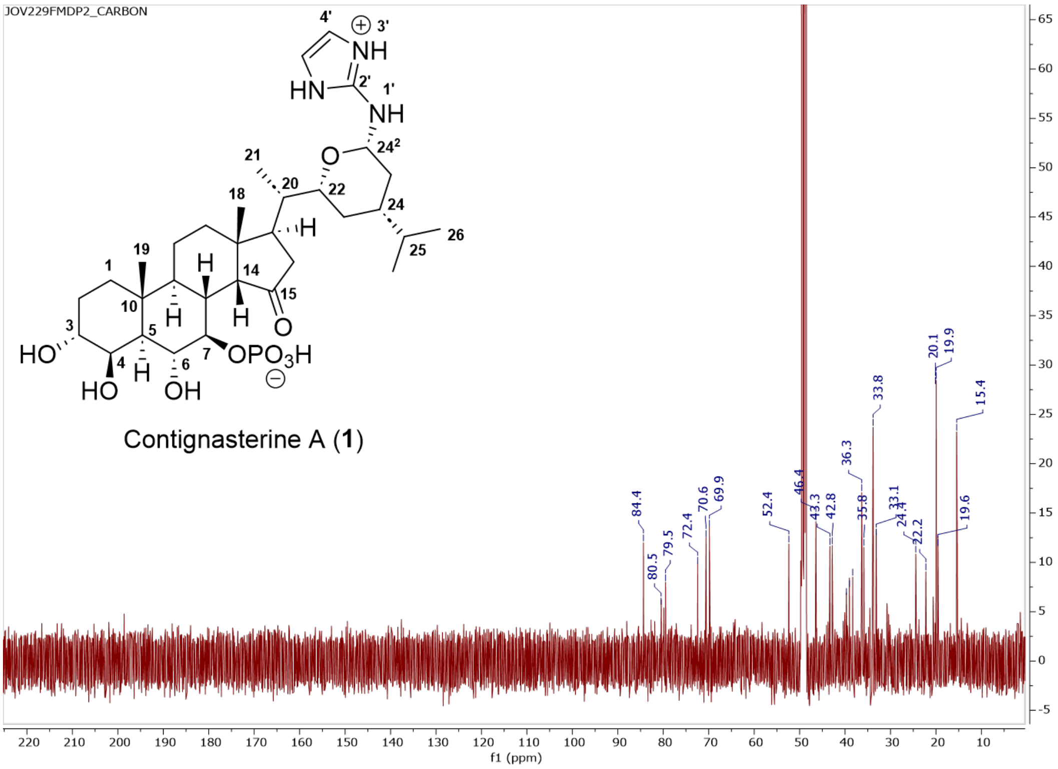

Figure S 6. ^{13}C NMR spectrum of **1** (CD_3OD, 150 MHz).

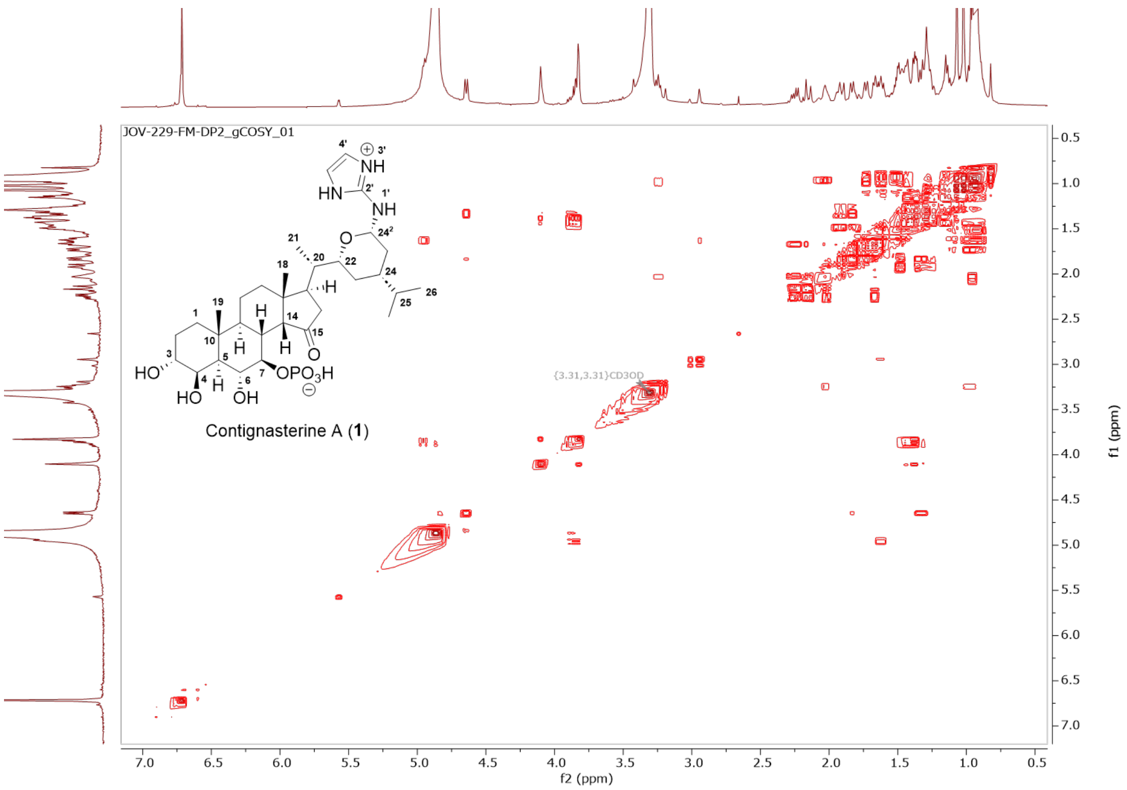

Figure S 7. COSY NMR spectrum of **1** (CD_3OD, 600 MHz).

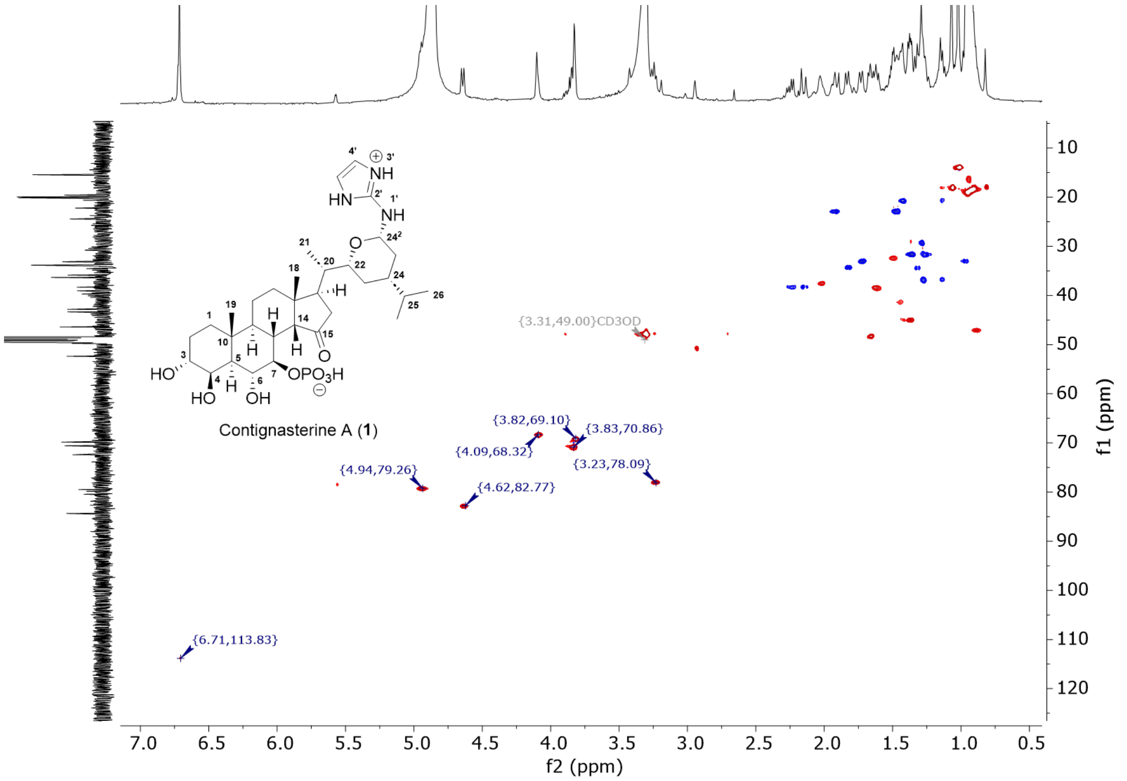

Figure S 8. HSQC NMR spectrum of **1** (CD_3OD, 600 MHz).

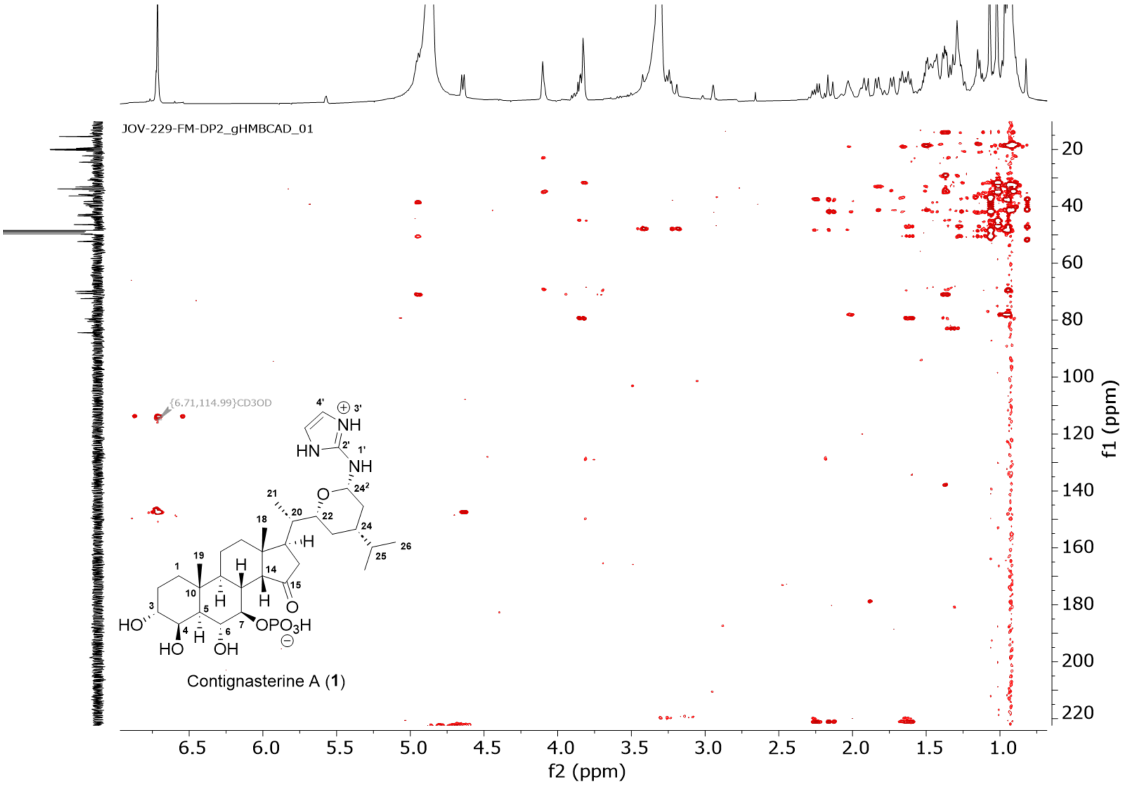

Figure S 9. HMBC NMR spectrum of **1** (CD_3OD, 600 MHz).

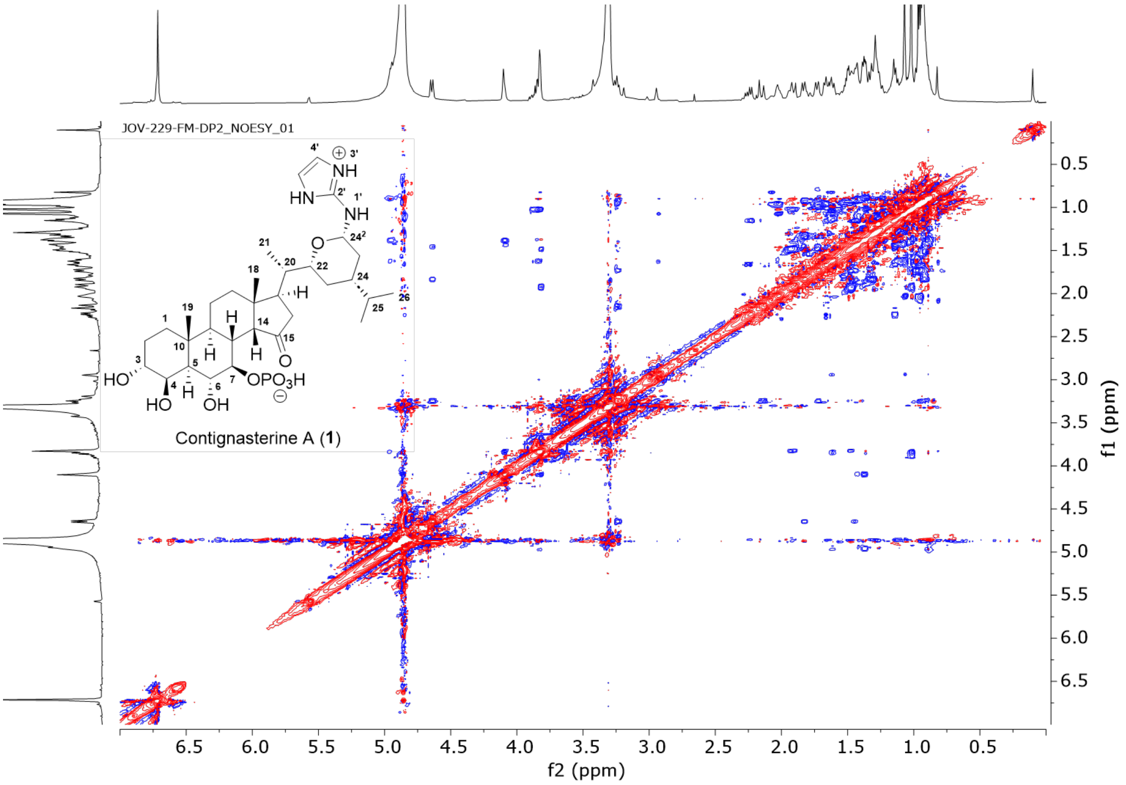

Figure S 10. NOESY NMR spectrum of **1** (CD_3OD, 600 MHz).

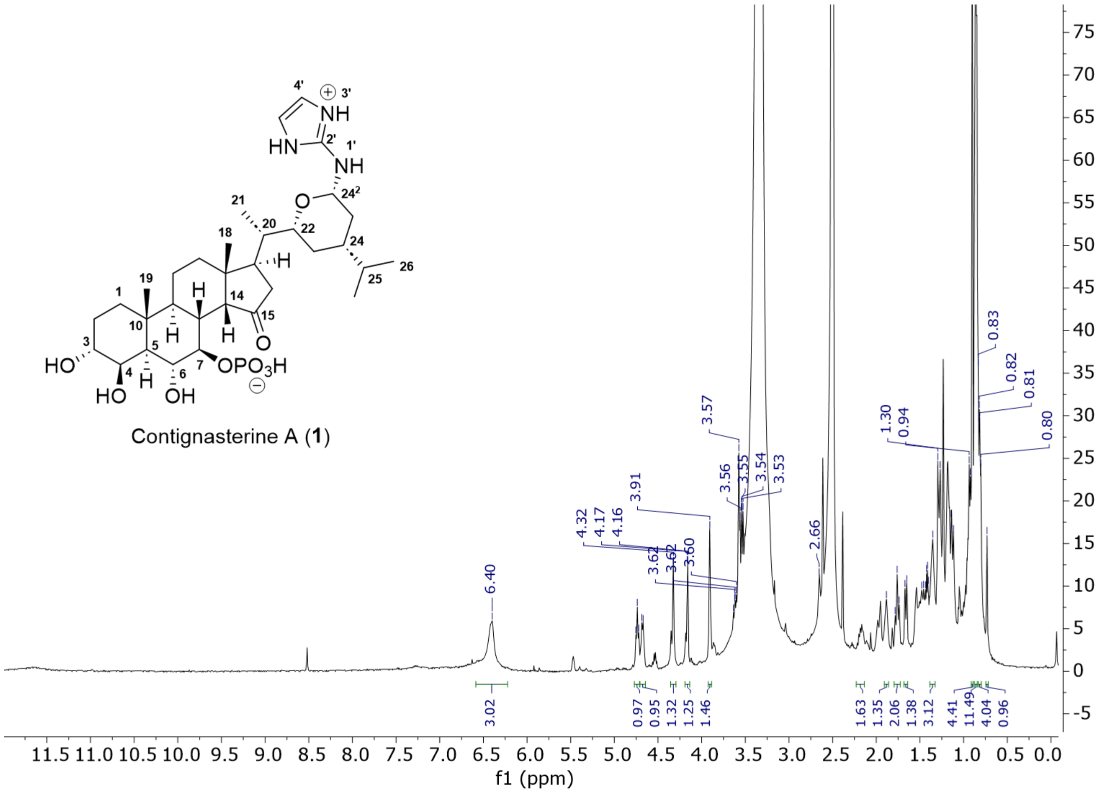

Figure S 11. ^{1}H NMR spectrum of **1** (DMSO-d_6, 600 MHz).

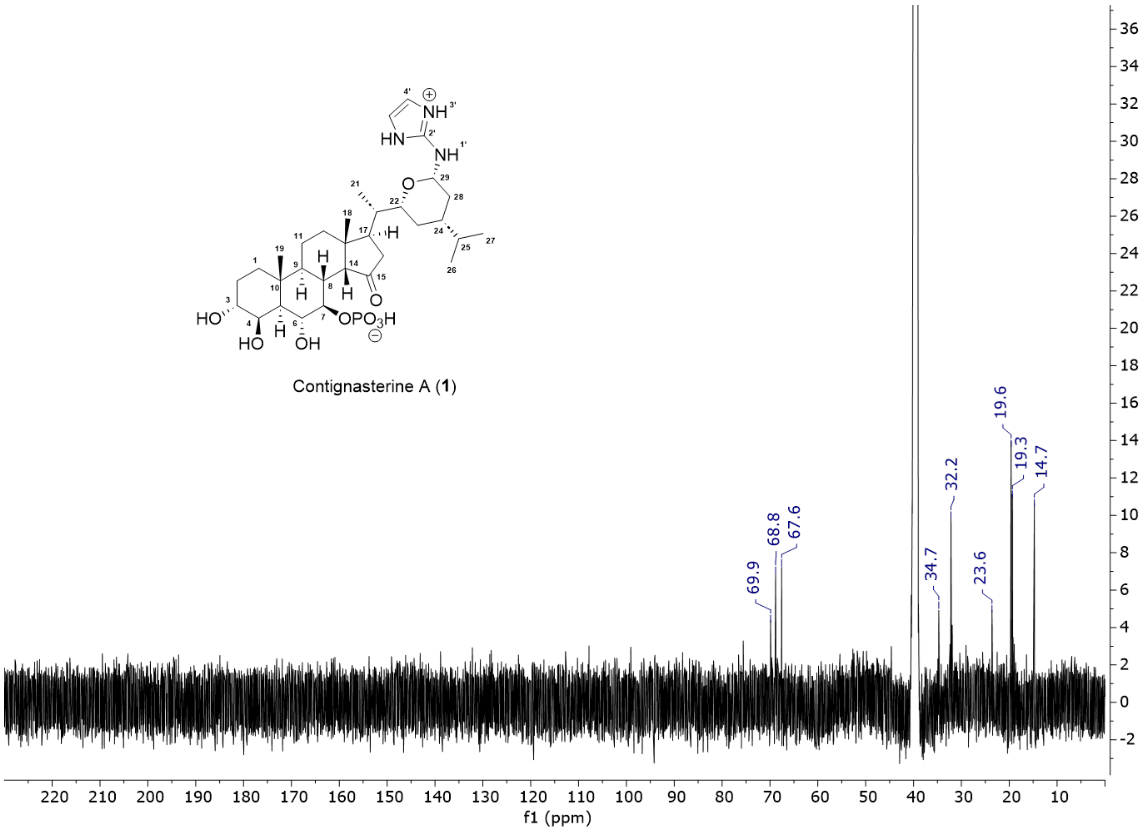

Figure S 12. ^{13}C NMR spectrum of **1** (DMSO-d_6, 150 MHz).

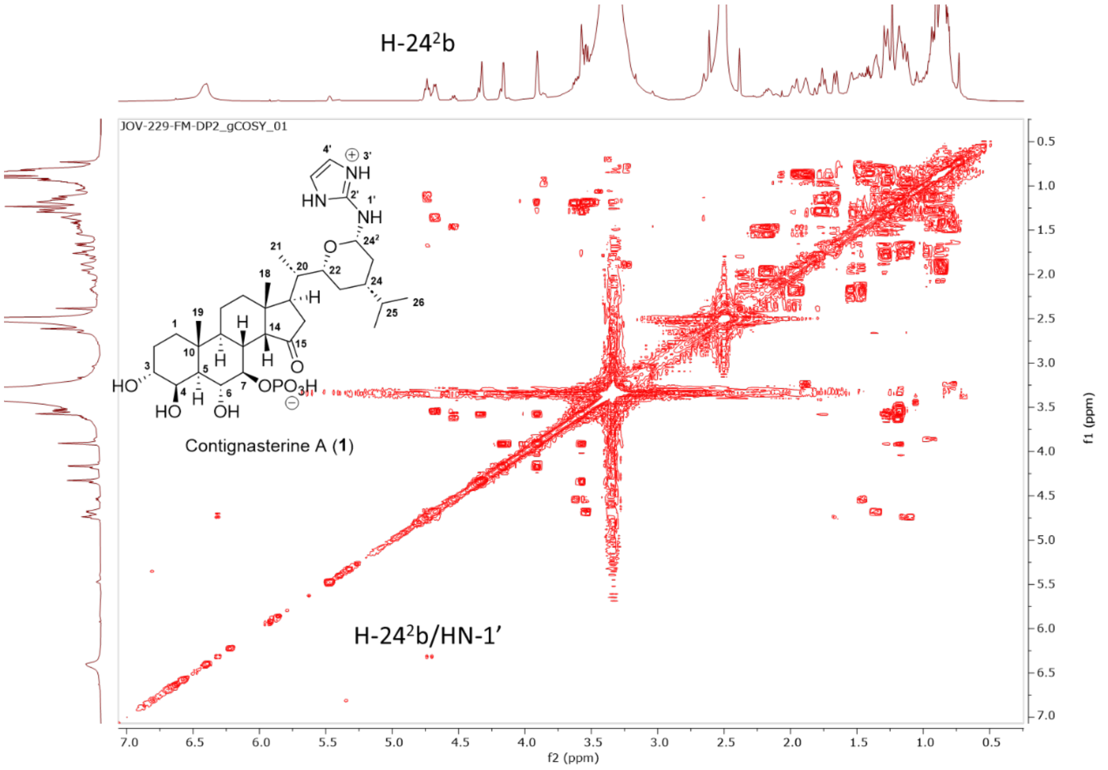

Figure S 13. COSY NMR spectrum of **1** (DMSO-d_6, 600 MHz).

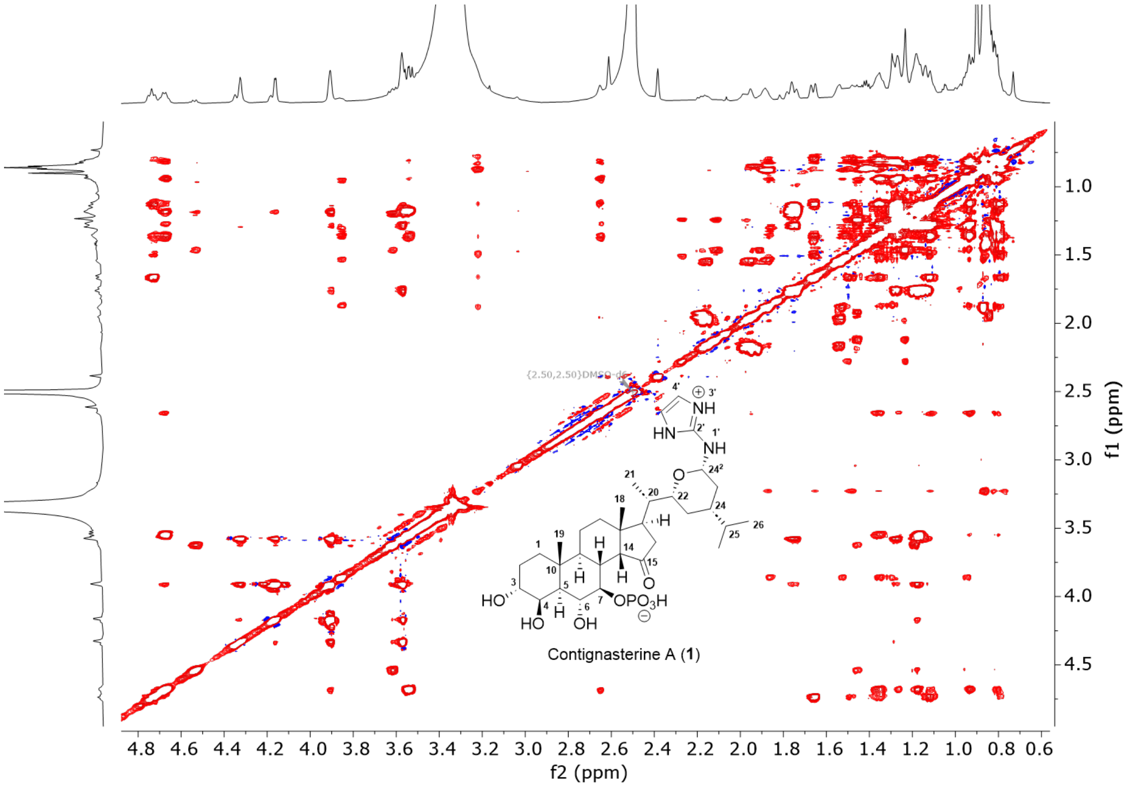

Figure S 14. NOESY NMR spectrum of **1** (DMSO-d_6, 600 MHz).

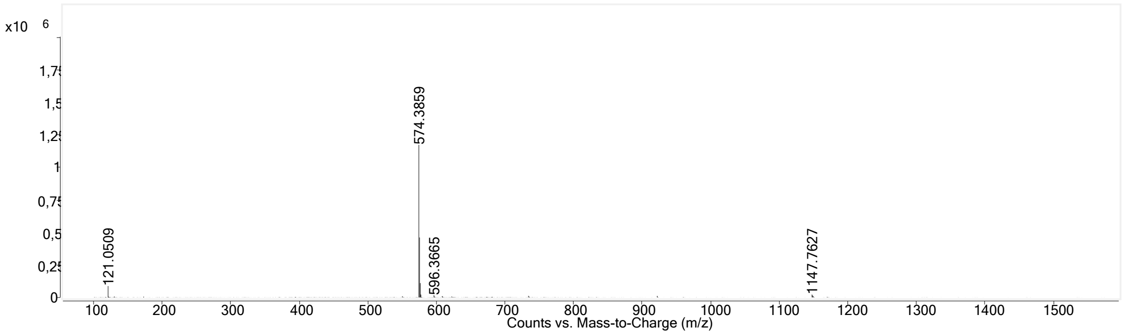

Figure S 15. (+)-HRESIMS analysis of compound **2.**

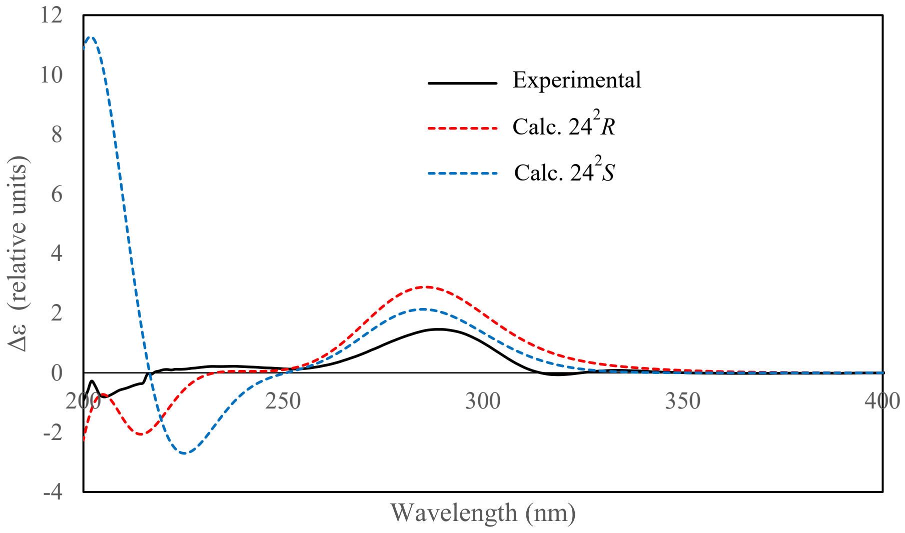

Figure S 16. Comparison of ECD spectra for **2** in CH_3OH.

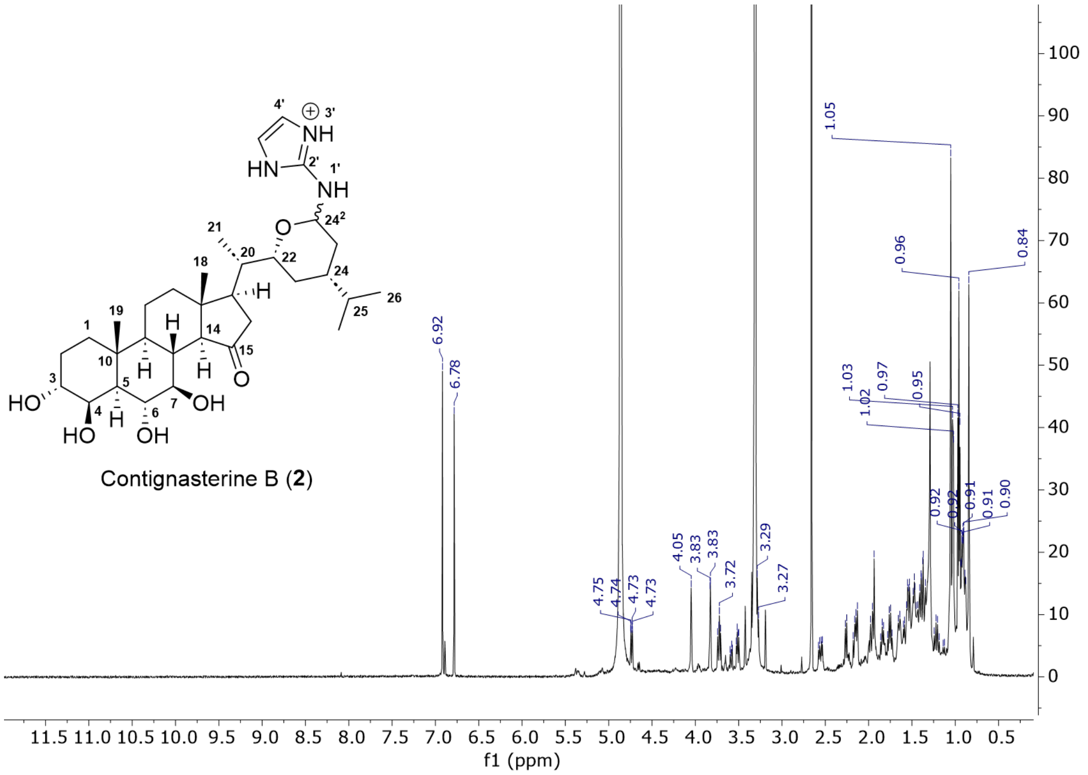

Figure S 17. ^{1}H NMR spectrum of **2** (CD_3OD, 600 MHz).

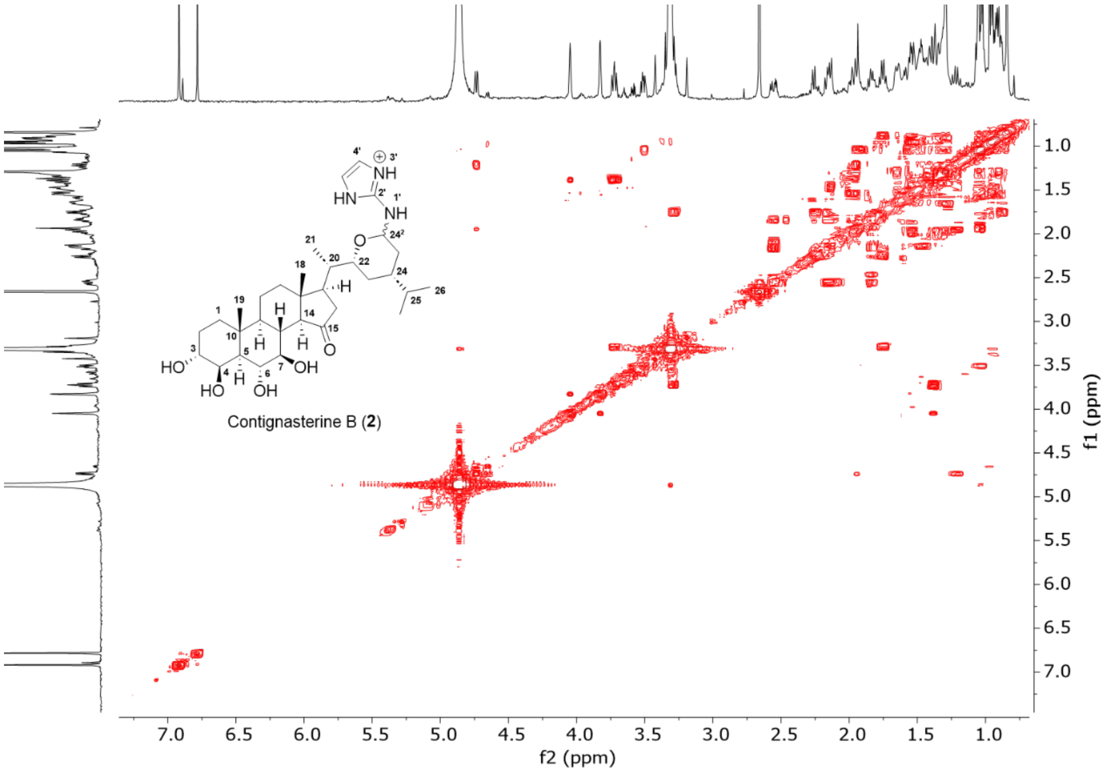

Figure S 18. COSY NMR spectrum of **2** (CD_3OD, 600 MHz).

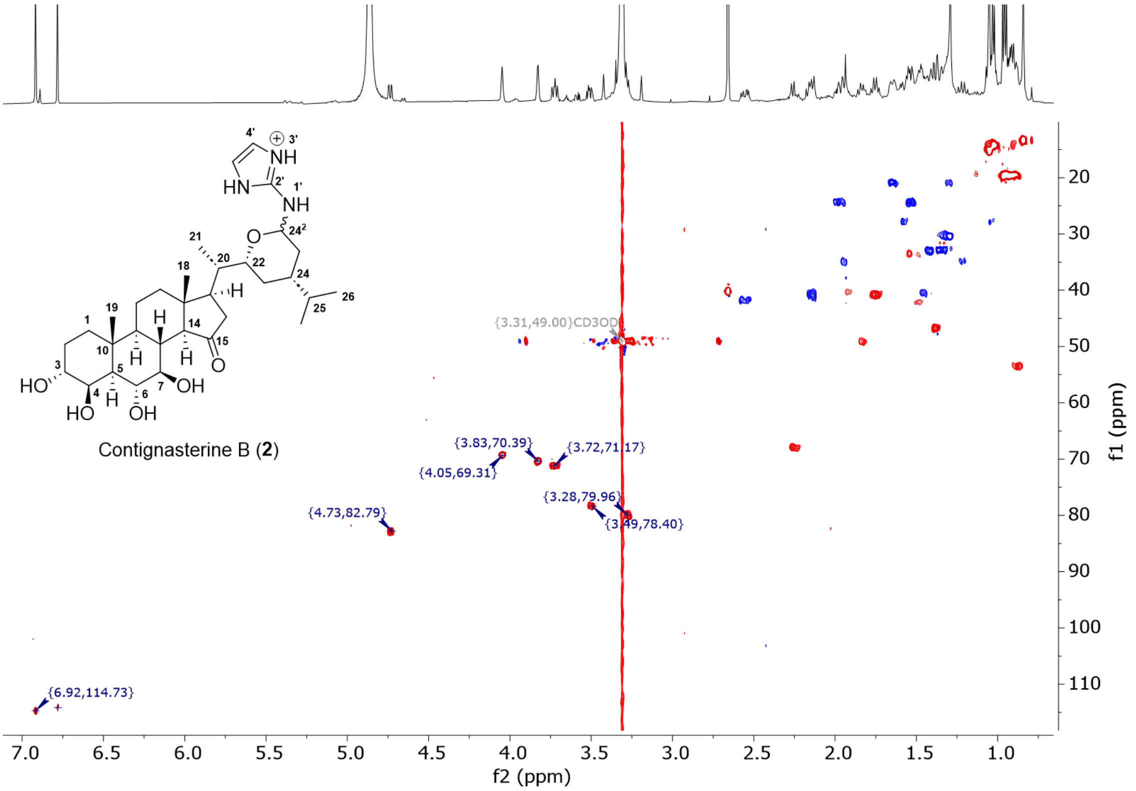

Figure S 19. HSQC NMR spectrum of **2** (CD_3OD, 600 MHz).

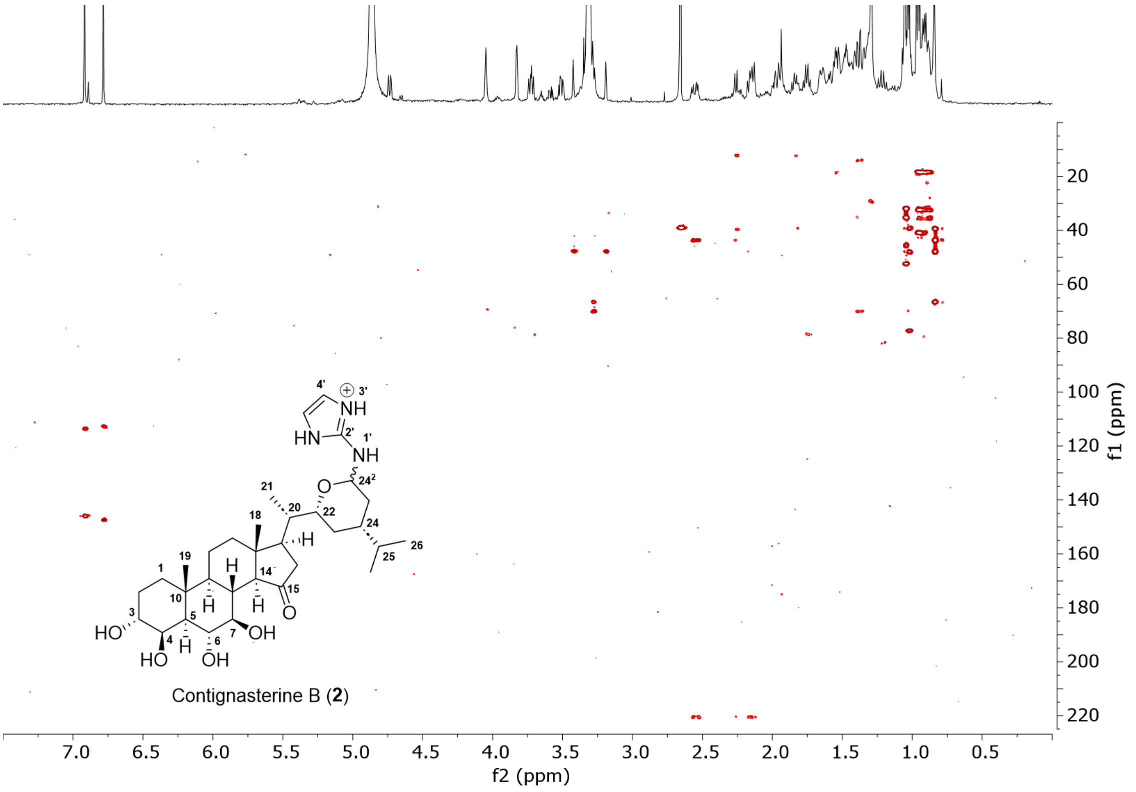

Figure S 20. HMBC NMR spectrum of **2** (CD_3OD, 600 MHz).

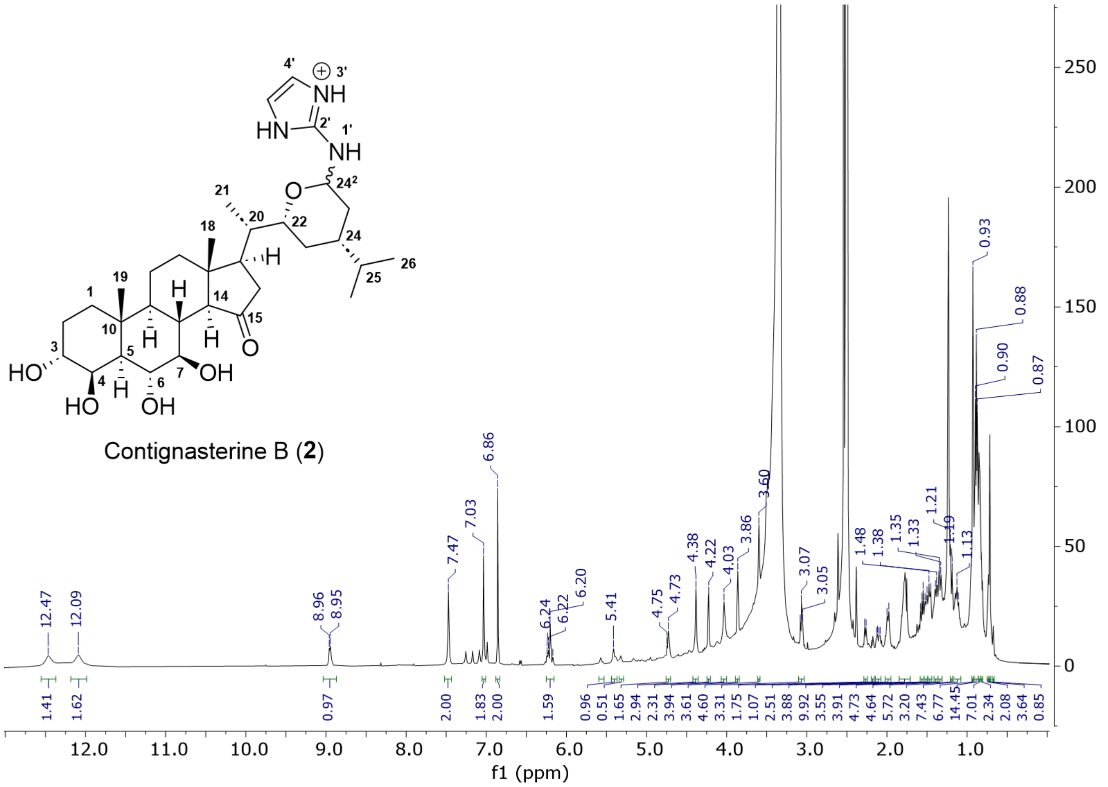

Figure S 21. ^{1}H NMR spectrum of **2** (DMSO-d_6, 600 MHz).

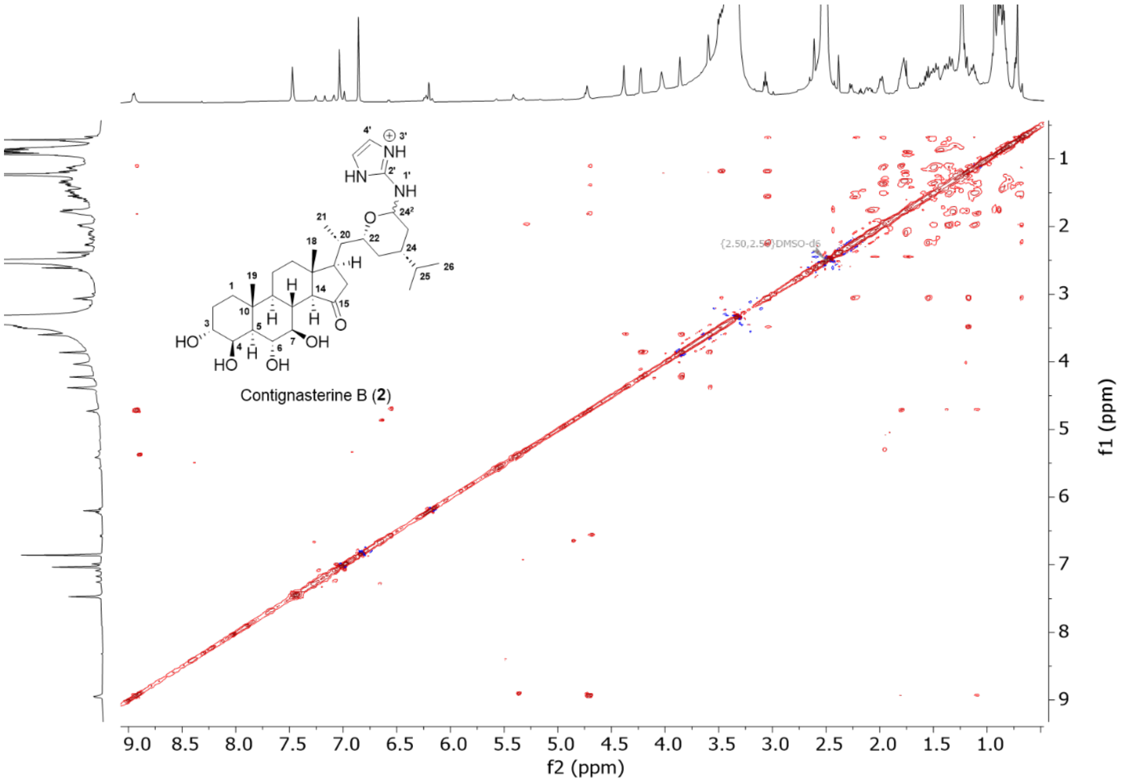

Figure S 22. COSY NMR spectrum of **2** (DMSO-d_6, 600 MHz).

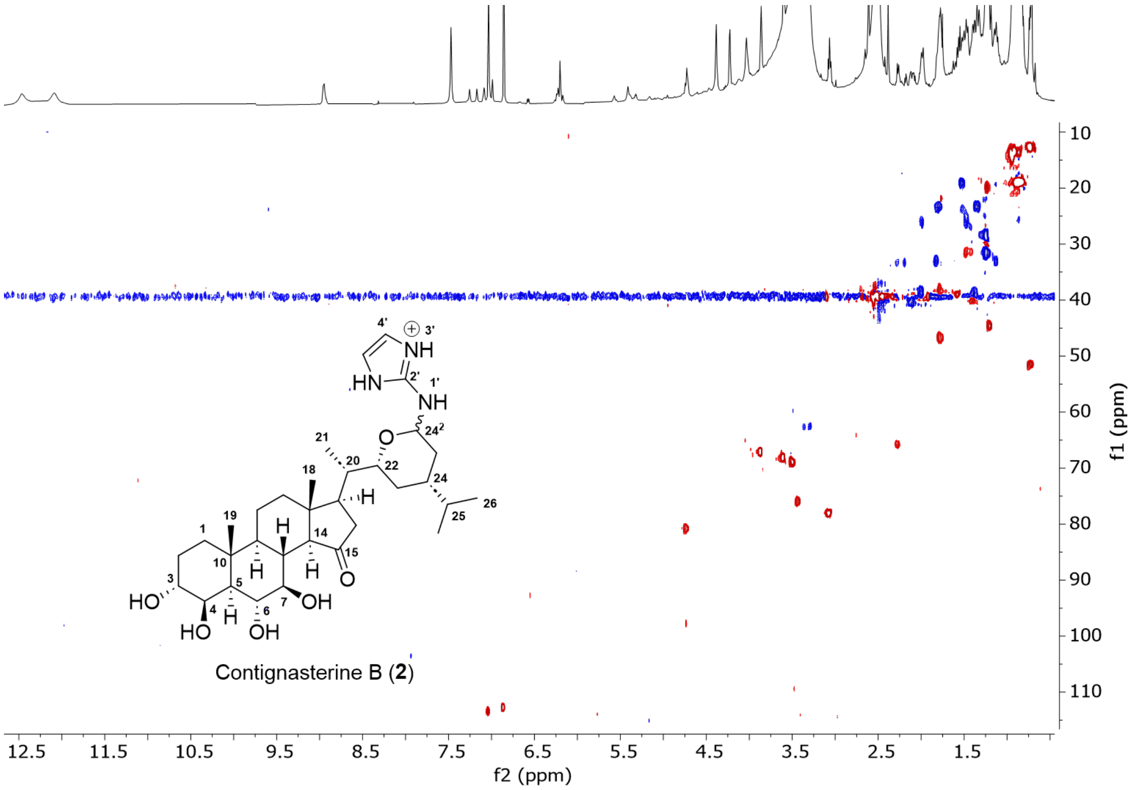

Figure S 23. HSQC NMR spectrum of **2** (DMSO-d_6, 600 MHz).

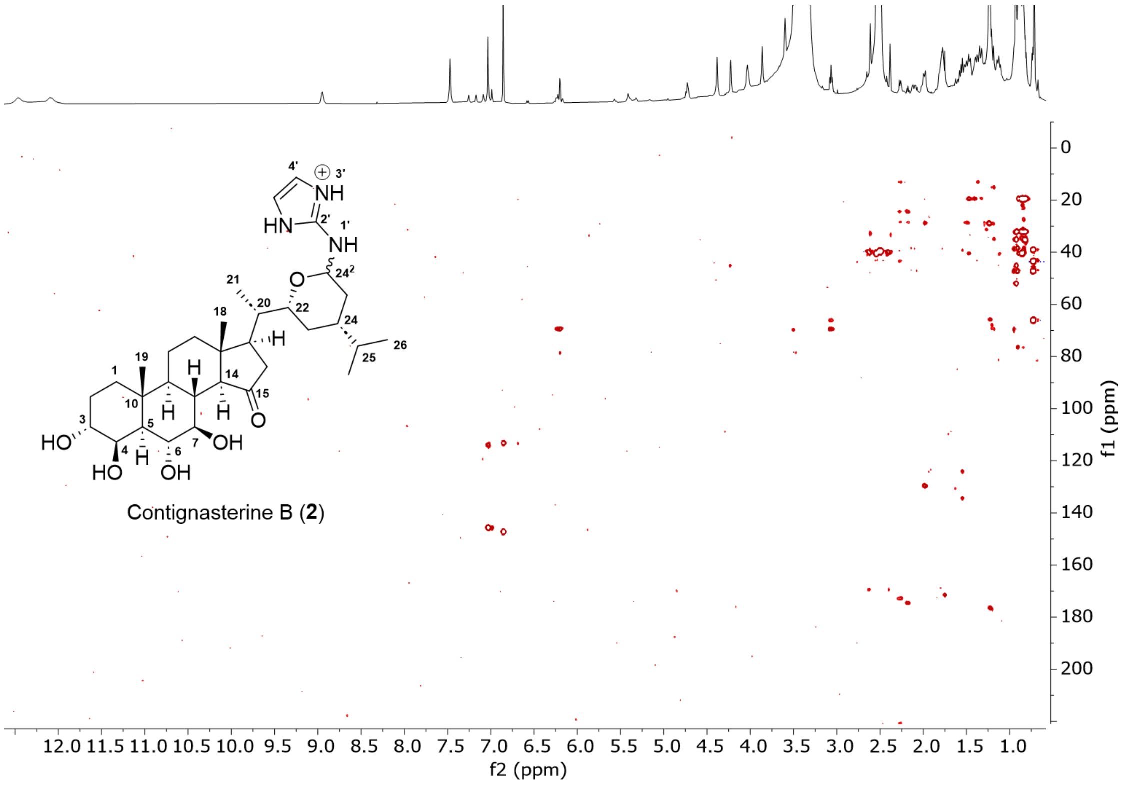

Figure S 24. HMBC NMR spectrum of **2** (DMSO-d_6, 600 MHz).

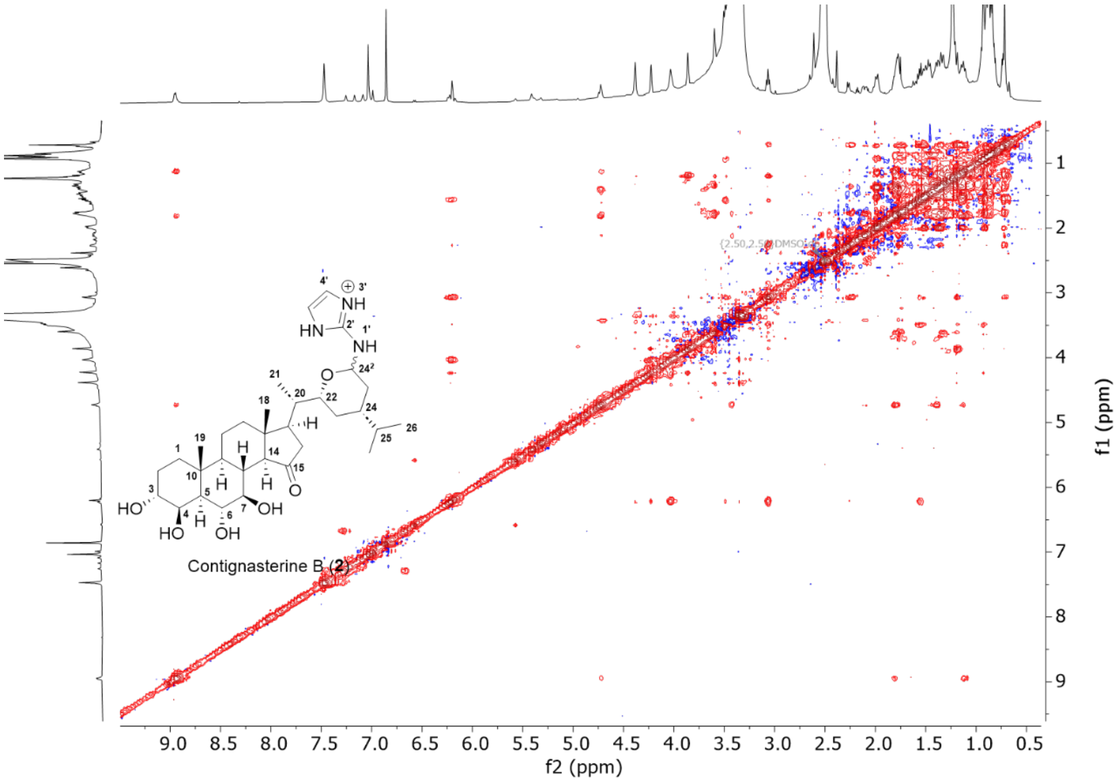

Figure S 25. NOESY NMR spectrum of **2** (DMSO-d_6, 600 MHz).

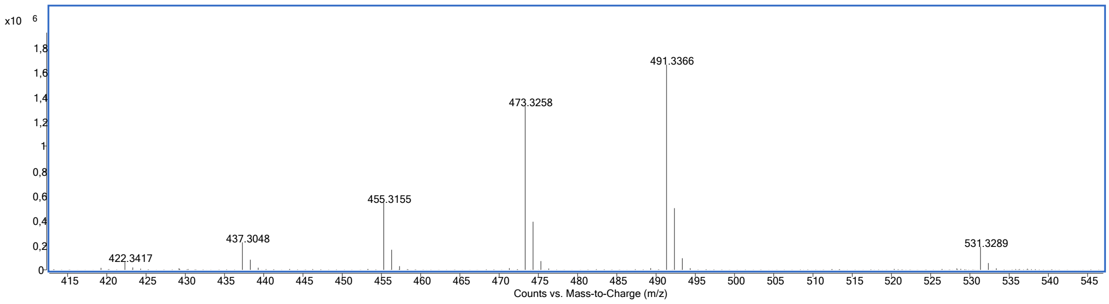

Figure S 26. (+)-HRESIMS analysis of compound **3.**

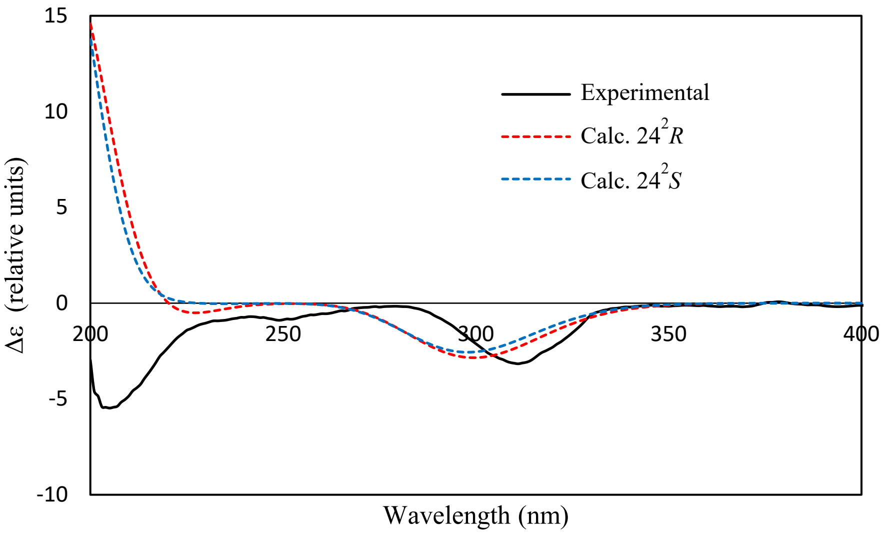

Figure S 27. Comparison of ECD spectra of **3** in CH_3OH.

Table S 1 NMR data for 24^2S and 24^2R epimers of contignasterol (**3**) in DMSO-d_6 (^{1}H 600 MHz and ^{13}C 150 MHz).

Position	24^2S- **3**		24^2R- **3**	
	δ_C, type	δ_H, mult. (J in Hz)	δ_C, type	δ_H, mult. (J in Hz)
1a	31.9, CH_2	1.17, br d (12.5)	31.9, CH_2	1.17, br d (12.5)
1b		1.24, m		1.24, m
2a	23.5, CH_2	1.31, m	23.5, CH_2	1.31, m
2b		1.75, m		1.75, m
3	68.4, CH	3.61, br s	68.4, CH	3.61, br s
4	67.2, CH	3.87, br s	67.2, CH	3.87, br s
5	44.8, CH	1.18, m	44.8, CH	1.18, m
6	69.9, CH	3.31*	69.9, CH	3.31*
7	73.4, CH	4.15, m	73.4, CH	4.15, m
8	38.1, CH	1.44, m	38.1, CH	1.44, m
9	46.0, CH	0.81, m	46.0, CH	0.81, m
10	35.6, C	-	35.6, C	-
11	20.1, CH_2	1.35, m	20.1, CH_2	1.35, m
12a	36.6, CH_2	1.10, m	36.6, CH_2	1.10, m
12b		1.29, m		1.29, m
13	41.4, C	-	41.4, C	-
14	50.5, CH	3.02, br s	50.3, CH	2.97, br s
15	219.6, C	-	219.4, C	-
16a	38.4, CH_2	2.10, br d (19.5)	38.4, CH_2	2.10, br d (19.5)
16b		2.42*		2.35*
17	45.3, CH	1.89, m	45.6, CH	1.83, m
18	18.8, CH_3	1.12, s	18.8, CH_3	1.12, s
19	14.7, CH_3	0.93, s	14.7, CH_3	0.93, s
20	39.5, CH	1.92, m	38.6, CH	1.84, m
21	16.5, CH_3	0.89, d (7.0)	16.5, CH_3	0.88, d (7.0)
22	75.0, CH	3.23, br t (9.5)	67.7, CH	3.79, br t (9.5)
23a	31.8, CH_2	0.73, m	32.4, CH_2	0.81, m
23b		1.51, m		1.56, d (12.0)
24	39.6, CH	1.31, m	34.5, CH	1.59, m
25	31.7, CH	1.40, m	31.7, CH	1.32, m
26	19.4, CH_3	0.83, d (6.0)	19.2, CH_3	0.81, d (6.0)
27	19.4, CH_3	0.83, d (6.0)	19.2, CH_3	0.81, d (6.0)
24^1a	35.6, CH_2	0.87, m	31.6, CH_2	1.16, m
24^1b		1.63, d (12.5)		1.52, m
24^2	95.1, CH	4.47, m	89.8, CH	5.16, br s
3-OH		4.36, d (3.0)		4.36, d (3.0)
4-OH		4.18, br s		4.18, br s
6-OH		4.05, br s		4.05, br s
7-OH		4.53, dd (18.0, 5.0)		4.53, dd (18.0, 5.0)
24^2-OH		5.97, d (5.0)		5.76, d (3.5)

*Overlapped with solvent or water signal.

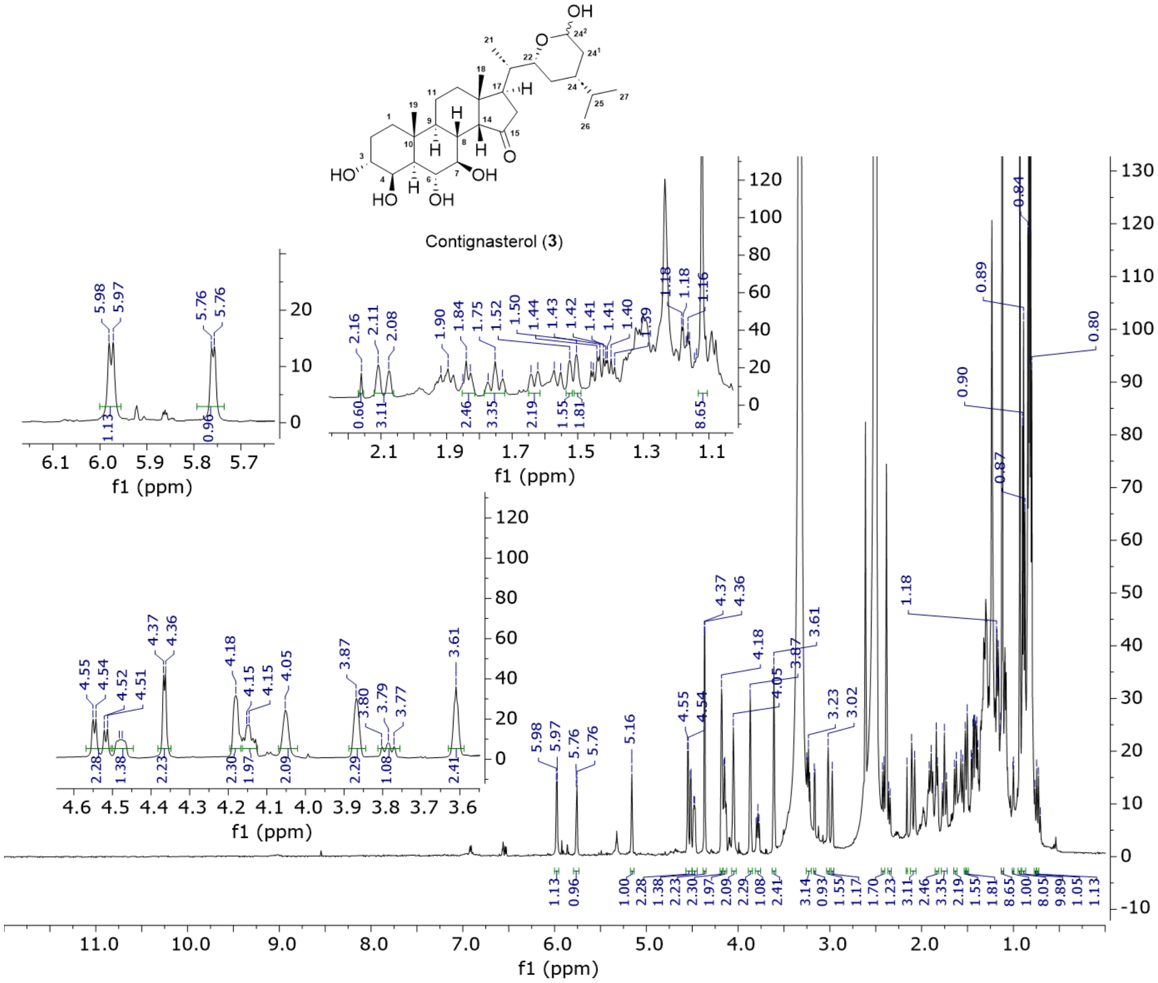

Figure S 28. ^{1}H NMR spectrum of **3** (DMSO-d_6, 600 MHz).

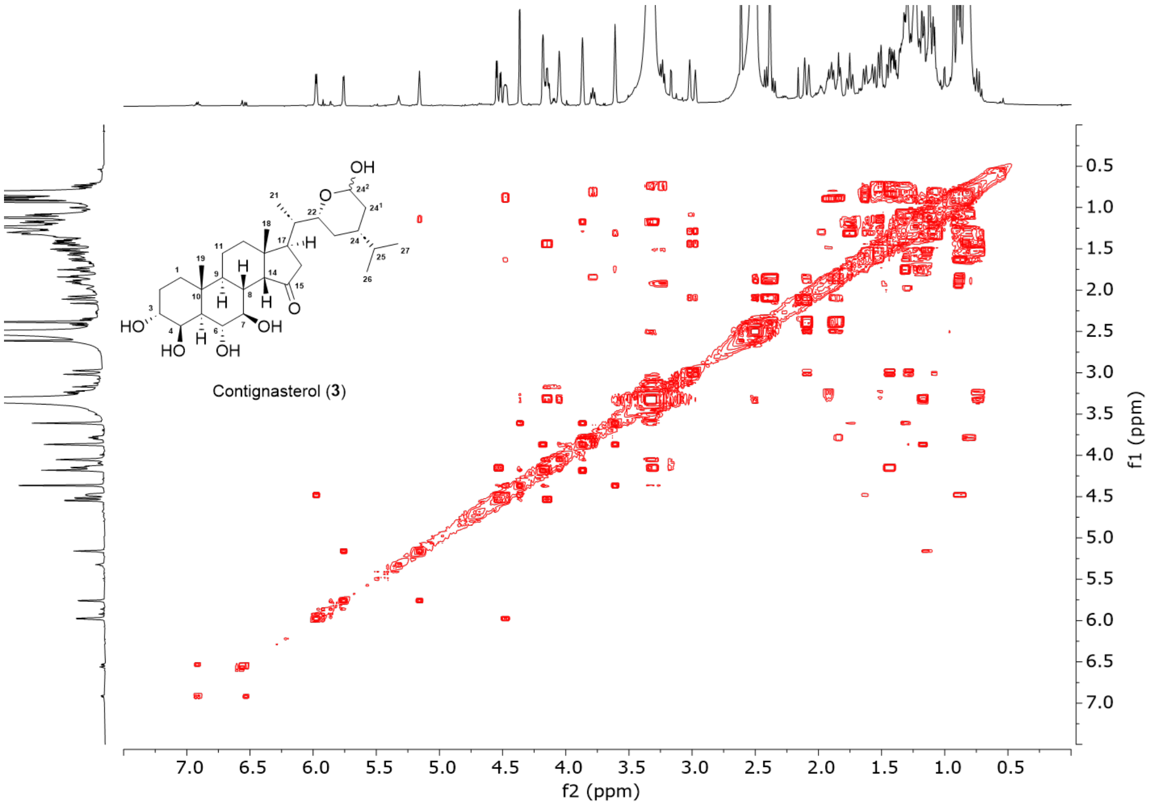

Figure S 29. COSY NMR spectrum of **3** (DMSO-d_6, 600 MHz).

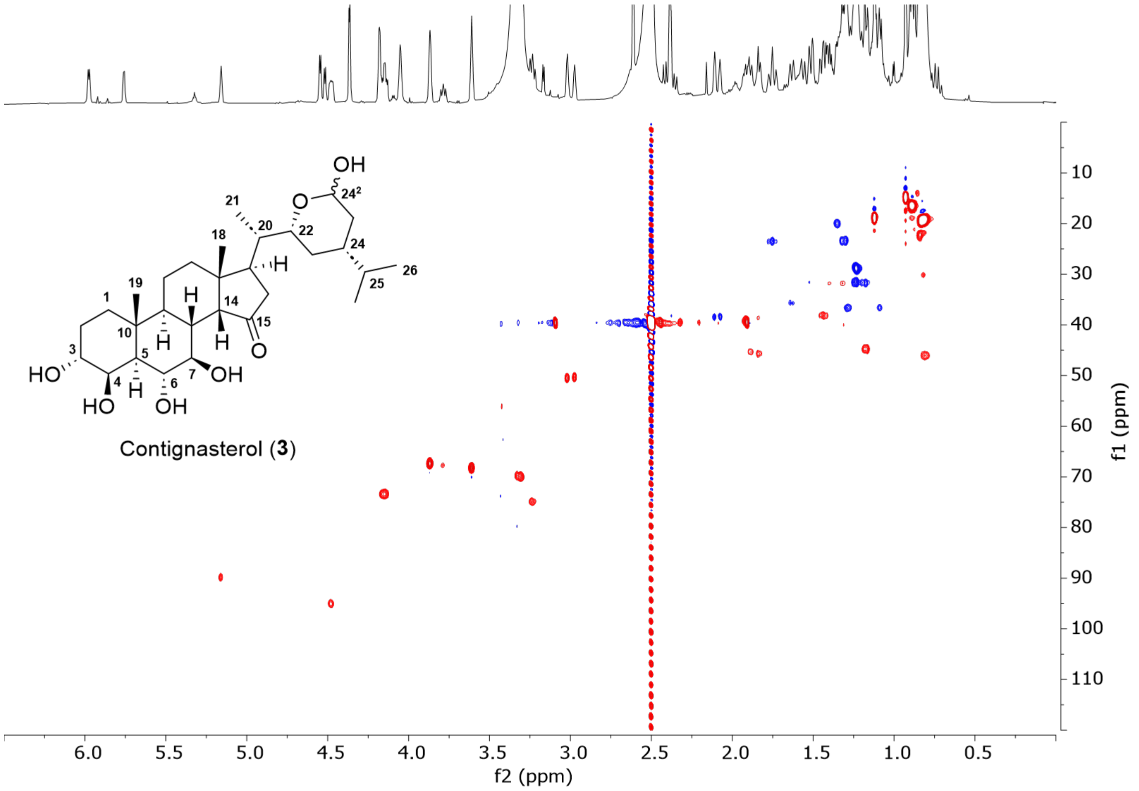

Figure S 30. HSQC NMR spectrum of **3** (DMSO-d_6, 600 MHz).

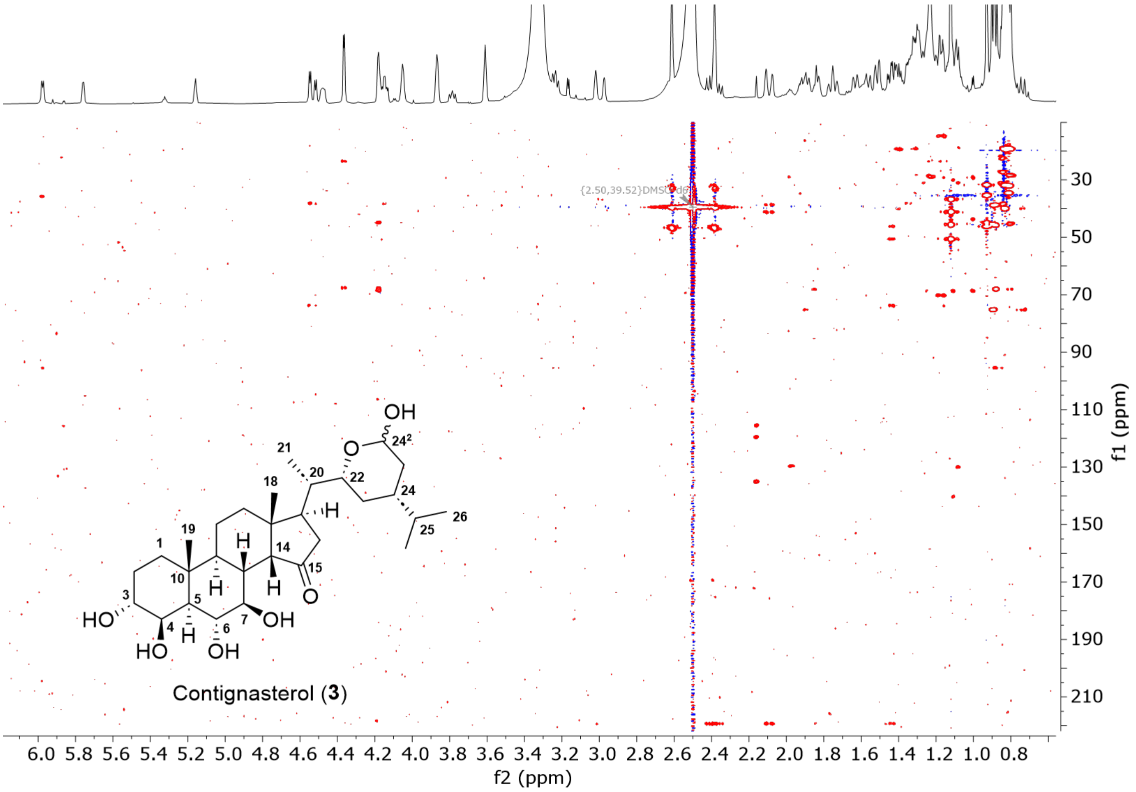

Figure S 31. HMBC NMR spectrum of **3** (DMSO-d_6, 600 MHz).

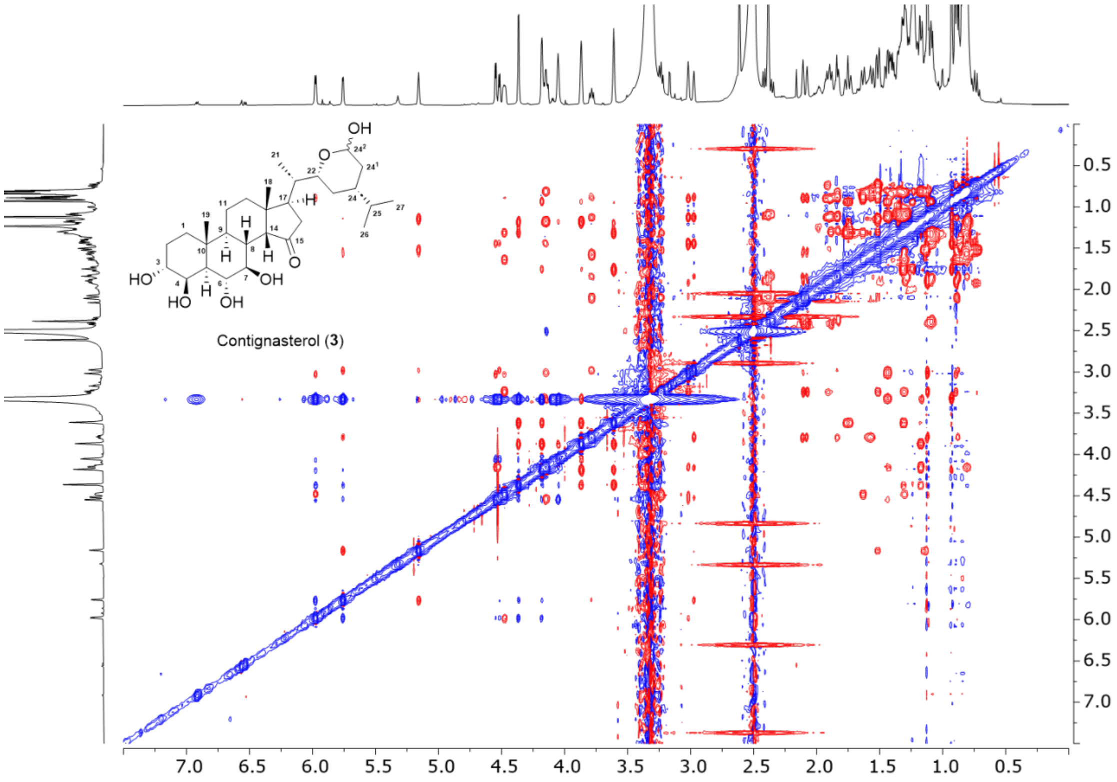

Figure S 32. ROESY NMR spectrum of **3** (DMSO-d_6, 600 MHz).
